# Prediction of binding poses to FXR using multi-targeted docking combined with molecular dynamics and enhanced sampling

**DOI:** 10.1007/s10822-017-0074-x

**Published:** 2017-10-20

**Authors:** Soumendranath Bhakat, Emil Åberg, Pär Söderhjelm

**Affiliations:** Division of Biophysical Chemistry, Lund University, Chemical Center, P. O. B. 124, 22100 Lund, Sweden

**Keywords:** D3R, Protein–ligand binding, Docking, Pose prediction, MD simulation, Reconnaissance metadynamics

## Abstract

Advanced molecular docking methods often aim at capturing the flexibility of the protein upon binding to the ligand. In this study, we investigate whether instead a simple rigid docking method can be applied, if combined with multiple target structures to model the backbone flexibility and molecular dynamics simulations to model the sidechain and ligand flexibility. The methods are tested for the binding of 35 ligands to FXR as part of the first stage of the *Drug Design Data Resource (D3R) Grand Challenge 2* blind challenge. The results show that the multiple-target docking protocol performs surprisingly well, with correct poses found for 21 of the ligands. MD simulations started on the docked structures are remarkably stable, but show almost no tendency of refining the structure closer to the experimentally found binding pose. Reconnaissance metadynamics enhances the exploration of new binding poses, but additional collective variables involving the protein are needed to exploit the full potential of the method.

**Electronic supplementary material:**

The online version of this article (doi:10.1007/s10822-017-0074-x) contains supplementary material, which is available to authorized users.

## Introduction

The binding of a small molecule (ligand) to a macromolecule (usually a protein) is a key process in biology and the most common mechanism for pharmaceutical drugs to act. A long-term goal of computational chemistry has been to accurately predict the binding free energy of a given protein–ligand pair. Several rigorous methods have been developed, which in principle are only limited by the accuracy of the underlying energy model (force field). These methods are typically based on molecular dynamics (MD) and include potential-of-mean-force methods, in which the ligand is physically dragged in or out of the binding cavity, as well as alchemical perturbation methods, in which an unphysical path is applied.

In practice, most of these methods require the knowledge of the structure of the protein–ligand complex, the *binding pose*, to converge in a reasonable time [1]. Thus, predicting an unknown binding pose is an important computational challenge in itself. The most common approach, *molecular docking* (or simply *docking*), typically involves a combination of a conformational search method and a *scoring function* that approximates the binding affinity for a given candidate. The receptor is usually considered rigid and the ligand flexible. However, some methods take the flexibility of the side chains in the receptor into account [2–4], for example by using “soft” scoring functions, which tolerates some overlap between the ligand and the protein [5, 6], or scanning rotamer libraries to simulate side chain movements [7].

Given the limitations of both conformational search methods and scoring functions, there is a growing interest in more rigorous approaches to the binding pose prediction problem. The direct use of MD simulations to find the binding pose has been tested for several systems [8–10]. In principle this approach can take into account both sidechain and backbone movements, but very long simulation times and multiple runs are typically required to obtain statistically valid results. Various types of enhanced-sampling methods have been applied to the problem to decrease the computational cost. One example is *reconnaissance metadynamics*, which uses a machine-learning approach to apply a local bias potential that helps escaping the free-energy basins in the conformational space [11]. In a previous work, we used reconnaissance metadynamics to find favorable binding poses for a rigid ligand binding to trypsin [12]. Methods capable of handling more flexible ligands have been developed based on other enhanced-sampling approaches, such as replica-exchange MD [13].

A more common application of MD simulations is to validate a binding pose obtained by docking [14]. The purpose of such investigations ranges from only confirming the kinetic stability of the pose in MD simulations [15], to estimating the binding free energy through approximate expressions like Molecular mechanics with Poisson–Boltzmann (or Generalized Born) and surface-area solvation (MM-PBSA or MM-GBSA) [16], or linear interaction energy (LIE) [17]. The results from such studied have been mixed and highly system-dependent [18–20]. Enhanced sampling has sometimes also been used to improve docking poses [21, 22], as well as to more thoroughly investigate the binding free energy landscape for a single ligand [23].

To assess the isolated pose-prediction problem, a crystal structure of the complex is needed, but then, on the other hand, there is a risk that studies get influenced by this knowledge. Therefore, blind challenges, in which there exists accurate experimental data for validation but the data is kept secret during the prediction phase, play a very important role. The first *Drug Design Data Resource* (D3R) Grand Challenge was conducted in 2015, with a first stage dedicated to pose prediction and ability to rank compounds by binding affinity with minimal structural data, and a second stage dedicated to ranking compounds when at least a subset of the binding poses were known. A conclusion from the challenge was that the accuracy of pose-prediction methods depends on several extrinsic factors, such as which protein structure was used for the docking, how protein structures were prepared, and other aspects of the protocol [24]. A second, similar challenge involving a new data set, *D3R Grand Challenge 2*, was initiated in 2016. In the first stage of this challenge, the goal was to determine the binding pose for 36 different ligands to the *farnesoid X receptor* (FXR) with computational methods. FXR is a ligand-activated transcription factor, attributed to many bodily functions, e.g. regulation and maintenance of bile acid synthesis, reduction of plasma cholesterol and triglycerides, glucose homeostasis and improvement of insulin sensitivity [25].

The aim of our participation in the D3R Grand Challenge 2 is to investigate whether rigid docking into a multitude of crystal structures, followed by extensive MD simulations, can solve some of the problems for which one would otherwise expect more advanced flexible docking methods to be required. In particular, we anticipate that the simulations, with their more accurate treatment of e.g. water, can refine resonable docking poses and bring them closer to the experimental structure. In light of previous research, we do not expect the MD simulations to be able to repair *mis-docked* poses in a reasonable amount of simulation time. Therefore we also include a third round of calculations, in which we apply an enhanced-sampling approach, namely reconnaissance metadynamics, to explore the generation of diverse binding pose candidates.

## Methods

### Overview

The pose prediction part of the D3R Grand Challenge 2 (which will simply be denoted the *challenge* in the following) involved predicting the binding pose of 36 ligands binding to FXR. One of the ligands (33) was subsequently discarded from the data set due to experimental problems; thus it will not be included in this manuscript.

After the submission of the blind predictions, we have continued the investigation to collect more statistics and get a more complete understanding of the merits and problems with the applied methods. In some cases, we have used the experimental data published after the submission deadline (which we will denote “secret data”) to analyze the results or guide the selection of computations to perform. However, because the aim of the study was to develop “blind” methods, we will clearly mention when and why the secret data was used.

### Preparation of the ligands

The preparation and parametrization of the ligands were done in a blind manner (i.e. without using secret data) and kept constant throughout the study. First, hydrogen atoms were added at pH 7.4 to the 2-dimensional molecular structures using Open Babel [26] followed by geometry optimization in vacuum using the MMFF94 force field [27, 28] with Open Babel’s obconformer tool. The antechamber [29] tool integrated with Amber 14 was utilized to parameterize the ligands with the Generalized Amber force field (GAFF) [30]. Partial charges were assigned using the AM1-BCC procedure [31]. Missing parameters were added by the automatic Amber tool parmchk2 without further optimization [32].

### Preparation of the proteins

An apo conformation of FXR was given at the start of the challenge, and was used as a template for the submission of binding poses. To account for the known conformational variation in the protein, a set of 18 ligand-bound crystal structures of FXR from the Protein Data Bank were prepared alongside the apo-protein (see Table S1 in the Supplementary material).

For each crystal structure, chain A was isolated by removing all water molecules as well as any redundant protein chains. To eliminate the dependence of the results on particular crystallographically motivated mutations in the various structures, all protein structures were modified by point mutations using the ’swapaa’ command in UCSF Chimera [33] until they had exactly the same sequence as the apo-protein (see Table S3 in the Supplementary material).

The apo structure was analyzed with the web server H++ [34], which predicted the protonation state for the receptor at pH 7.4. All histidines were found to be singly protonated on the $\hbox {N}_\epsilon$, and all other amino acids were in their typical protonation state. These protonation choices were then transferred to the other crystal structures, so that all the final prepared structures contained exactly the same set of atoms.

For the *a posteriori* analysis, a similar procedure was used to prepare the new (secret) crystal structures for simulations, as detailed in Table S3 in the Supplementary material [35, 36]. Owing to a greater crystallographic sequence variation, we did not enforce a completely identical sequence for these structures in the simulations.

### Docking

Molecular docking was performed by Autodock Vina, which is an open source docking programme widely used by many research groups for docking and virtual screening studies. It employs an empirical scoring function [37] which is inspired by X-score [38].

Autodock Vina uses a fixed grid box wherein it tries to place the different conformations of the ligand. In our case, the previously published crystal structures of FXR–ligand complexes indicated a single well-defined binding site. The AutoDock Tools [39] were used to create a grid box for each protein structure with a grid spacing of 1.0 Å, a size of 15 × 15 × 15 Å, and with the grid centered on the center of mass of the removed co-crystallized ligand.

The docking was performed with the “exhaustiveness” set to 8. Increasing the exhaustiveness would increase the probability of finding the global minimum, but we preferred to set it to a typically used value. As the docking procedure involves random seeds, different results are obtained in every run; we simply used the first set of results that we obtained.

Each ligand was docked with every crystal structure, including the apo-protein, in total 35 × 19 docking runs. After the secret data was revealed, we extended the docking study to include the new crystal structures. Again, each ligand was docked into all available crystal structures and all parameters of the docking protocol were kept identical.

### MD simulations

For each ligand, the top-predicted docking pose, i.e. the prediction with the best score among all included crystal structures, was used as the starting point for an MD simulation. The Amber *ff14SB* force field [40] was used for the protein and the GAFF force field was used for the ligand, as described above.

All simulations were run with GROMACS 4.6.2 [41]. The acpype script [42] was used to prepare GROMACS-compatible files. Using the standard GROMACS tools, the complex was placed in a truncated octahedron box, with a minimum distance from the complex to the box boundary of 8 Å, and solvated with water modeled by the TIP3P force field. Energy minimization was performed for each complex using a steepest-descent integrator for 200 steps. Each system contained one protein–ligand complex and $\sim$ 12000 water molecules.

A 1 ns NPT equilibration (constant composition, pressure, and temperature), with positional restraints on the $C_\alpha$ atoms of the receptor and the heavy atoms of the ligand (using a force constant of $120\,\hbox { kcal }\,\hbox { mol}^{-1}\,\hbox { nm}^2$), was performed to allow the water to relax around the complex. A non-bonded cut-off of 9 Å was used and the long-range electrostatics were treated using Particle mesh Ewald (PME) summation [43] using a grid spacing of 0.12 nm. The pressure was maintained at 1 bar using the Berendsen barostat. A leap-frog integrator algorithm with a time step of 2 fs was used, and all bond lengths were constrained using the LINCS algorithm. The temperature was kept constant at 310 K (i.e. the temperature in the human body) using the velocity–rescaling algorithm [44].

The production MD simulation was performed with the same settings as the previous equilibration, but without any restraints. In order to check the stability of the binding pose, an RMSD calculation was performed after each ns of MD simulation to see whether the ligand position deviated too much from the starting position. If the RMSD of the ligand (after alignment of the $\hbox {C}_\alpha$ atoms of the protein) at any frame exceeded 2.5 Å from the average structure of the first nanosecond of free simulation, or 4 Å from the starting pose, the simulation was terminated; otherwise it was run for 50 ns.

### Reconnaissance metadynamics simulations

Reconnaissance metadynamics (RMD) simulations were performed for all the complexes subjected to MD simulations, using the end points of the MD simulations as starting points for the RMD simulations. RMD is a self learning algorithm for enhanced sampling which is capable of handling a larger number of *collective variables* (CVs) than ordinary metadynamics and related methods [11]. The RMD calculations were performed using version 1.3 of the PLUMED plugin for free-energy calculations [45] patched with GROMACS.

The complete set of rotable bonds in each ligand was determined manually and the corresponding dihedrals were used as CVs in the RMD algorithm. The RMD simulations were performed with the same settings as the previous MD simulations except that they were performed in the NVT ensemble (constant composition, volume, and temperature) to avoid technical issues. The bias deposit stride was set to 1 ps with a gaussian width of 1.5 (in the dimensionless local metric defined by the covariance matrix) and a gaussian height of 0.239 kcal/mol. The basin tolerance was set to 0.2 the basin expand parameter to 0.3 and the basin initial size to 1.5. The RMD clustering stride was 100 ps, with 1000 data points collected during this period. RMD simulations were run for 20 ns and the results were interpreted by a clustering approach, as described below.

For some ligands, we also ran an RMD simulation with the ligand dihedrals and seven additional CVs intended to increase the fluctuation of the protein sidechains in the active site. These were the dihedral angle around the $C_\alpha$–$C_\beta$ bond for seven active-site side chains: Leu-291, Met-294, His-298, Met-332, Ser-336, Leu-352, and Ile-356.

For ligand 5, we also ran an RMD simulation with the ligand dihedrals and three additional CVs intended to promote rotation of the ligand with respect to the protein. These were again dihedral angles, but each connecting two $\hbox {C}_\alpha$ atoms in rigid parts of the protein with two atoms of the ligand. The atoms were manually selected by visual inspection of the docked binding pose, in order to represent three different modes of rotation of the ligand (see Fig. S1 in the Supplementary material for details).

### RMSD analysis and clustering

Two types of RMSD analysis were performed in this study. To analyze the difference between various poses, perform clustering, and analyze the stability of a simulation, the standard GROMACS tools were used, with the RMSD calculated for the heavy atoms of the ligand after alignment of the $C_\alpha$ atoms of the protein. To analyze the deviation from experiment, we instead used the official script provided by the D3R team, which takes into account symmetry-equivalent atoms by using the *maximum common substructure* procedure [46] to match ligand atoms between the prediction and the reference structure in such a way that the RMSD is minimized. Chain A of the experimental structures was used as the reference structure, except in some cases where a slightly lower RMSD was obtained if using chain C. If several alternative conformations were present in the reference structure, we used the set of coordinates that gave the lowest RMSD.

Cluster analysis was performed with the GROMACS tool g_cluster using the *GROMOS* algorithm [47] with the RMSD distance metric defined above. Snapshots from the trajectory were taken out with a period of 20 ps, the RMSD cut-off was set to 2.0 Å, and only clusters containing at least ten structures were considered to be significant.

#### Selection of poses for submission

For preparing the docking submission to the challenge, all poses for a given ligand were ranked according to their score, and any duplicate poses were removed by going through the sorted list of poses and discarding a pose if a similar pose with better score had already been selected, possibly based on another crystal structure. The criterion for similarity was that the RMSD between the poses was below 2 Å. The top five poses for each ligand were submitted in the challenge, ranked according to their score.

For preparing the MD submission to the challenge, the clusters of the MD trajectory were ranked according to size (i.e. the number of snapshots). The center of the largest cluster was used as the top pose. In most cases, only one significant cluster was obtained, and in the few cases in which several (up to four) significant clusters were obtained, the extra clusters were found to be uninteresting and will not be further discussed.

When preparing the RMD submission to the challenge, only three ligands were ready. The poses for these ligands were selected by manually combining data from RMD and additional MD simulations, as described in Table S2 in the Supplementary material. For all other ligands, the MD submission was reused.

## Results and discussion

For computationally predicting binding poses to FXR, we investigated the performance of a rigid but multi-targeted docking method and further refinement by MD and enhanced sampling, all in a blind-challenge context provided by the D3R Grand challenge 2. Our long-term goal is to develop a useful combination of these methods which can be applied to protein–ligand complexes with unknown structure. The results will be presented and analyzed step-wise, beginning with docking results, continuing with results from MD simulations, and ending with the results from reconnaissance metadynamics.

### Docking results

To account for the known flexibility of FXR and increase the probability of finding the correct binding pose despite using a rigid docking method, each ligand was docked into a series of crystal structures from the Protein data bank, as described in the method section. In the analysis stage, this set of protein structures was further extended by the 35 new crystal structures and a similar docking procedure was performed.

Our docking submission (*0lxp5*) consisted of five predicted poses for each ligand, ranked according to their score. The evaluation results (RMSD towards experiment) are shown in Table 1. The column “first” gives the RMSD for our top-predicted pose, whereas the column “best” gives the smallest RMSD among the five predicted poses. Both these measures were included in the official evaluation of the challenge; the “best” measure primarily tests a method’s ability to *find* the binding pose, whereas the “first” measure also tests the ability to *rank* the poses.

**Table 1:**
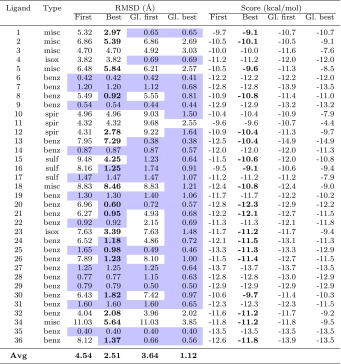
Results of the docking

Evaluation of the RMSD towards experiment for the top-predicted pose (first), best of the five submitted poses (best), best of all poses found in the analysis stage of the docking including the new crystal structures (globally best), and top-predicted pose when both old and new crystal structures were used (globally first). The docking score of each pose is also given. For improved readability, the entries for “best” are shown in bold face if they are not identical to “first”. The shaded table entries represent poses with an RMSD below 2 Å. The ligands are classified into types listed in Table 3

**Fig. 1:**
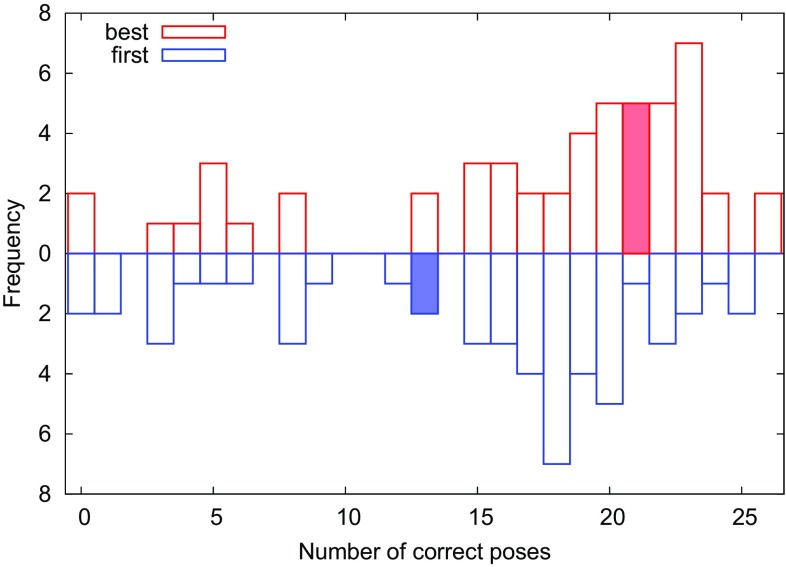
Performance of the submissions to D3RGC2. The upper panel shows the distribution of the number of correctly predicted ligand poses (out of 35) over all the submissions, if the *best* of up to five poses was considered. The lower panel shows the corresponding results when only the *first* (top-predicted) pose was considered. The filled rectangles show the performance of our docking submission. Any pose with RMSD less than 2 Å towards the crystal structure was classified as correct

Using an RMSD threshold of 2 Å for classifying a pose as “correct”, the docking procedure included the correct pose among the five submitted poses for 21 out of 35 ligands. However, only for 13 of these ligands, the correct pose was submitted as the “first” pose (i.e. having the best score), although for the remaining eight cases the difference in score was always smaller than 1 kcal/mol (0.44 kcal/mol on average; see Table 1). Statistics for other threshold values are given in Table 2 and show similar trends. The distribution of the number of correct poses over all D3RGC2 submissions are shown in Fig. 1 (data extracted from the official web server [48]).

The “globally best” column in Table 1 contains the results of the analysis stage, in which the experimental protein structure was included among the docking targets (i.e. secret data was used, but still no information about the ligand pose). In this case, 29 ligands were correctly docked (again with a threshold of 2 Å), i.e. including 8 ligands that were not correctly docked in the first stage. Thus, as expected, having the correct protein structure greatly helps when performing rigid docking. This is especially true for ligand types not represented in the set of old crystal structures. In fact, 6 of the 8 improved ligands belonged to the *isoxazoles*, *spirocycles*, and *miscellaneous* groups, and these groups also showed a substantial improvement in average RMSD when going from the first to the analysis stage, as shown in Table 3. However, the results do not reveal whether the major advantage comes from having a correct *global structure* of the protein (e.g. positions of helices) or from having the sidechains in exactly the correct conformation for optimal interaction. From the data in Table 2, we can conclude that the advantage increases if the threshold is decreased (e.g. it is even more important to have the correct protein structure if one aims at an RMSD below 1 Å), but this fact concurs with both the two explanations (global structure and sidechain conformations) and thus does not discriminate between them.

To analyze the performance of the scoring function, we posed the question of whether our multi-targeted docking approach would have picked out the correct pose if the challenge had included the secret crystal structures of the proteins (but no information about binding poses or which protein structure corresponded to the particular ligand). More precisely, we extracted the RMSD towards experiment for the single pose having the best score among all the dockings to old and new crystal structures. The results are given in Table 1, in the column “globally first”, and summarized in Table 2. Compared to the “first” results, which were obtained in the same manner but without the new crystal structures, the number of correctly predicted poses increased from 13 to 19. The discrepancy between this number (19) and the “globally best” (29) shows the difficulty for the scoring function to select the correct binding pose. The selection is actually slightly easier if, for each ligand, only its own protein structure is used for docking (22 correct poses; see Table S4 in the Supplementary material for details). However, it is noteworthy that this result is still worse than for some blind submissions to the challenge (cf. Fig 1). Moreover, in a real application, the exact protein structure is typically not known, and there appears to be no significant advantage of restricting the set of protein structures to those involving the same type of ligand (20 correct poses; see Table S4); in contrast, the inclusion of many structures is often beneficial because it reduces the method’s sensitivity to both experimental errors and scoring function deficiencies.

With this larger set of data, it is interesting to know whether the difference in docking score between the correct pose and the top-predicted but wrong pose is always small, as was indicated by the data for the old crystal structures (see above). We thus defined the correct pose as the globally best-scored pose with an RMSD below 2 Å, and excluded ligands for which no such pose was found. The detailed results are shown in Table S5 in the Supplementary material. Out of the 10 ligands for which the top-predicted pose was wrong, 5 displayed a score difference smaller than 0.5 kcal/mol, which can be considered well within the accuracy limit of the scoring function; one should probably consider all poses equally likely if their scores differ by such small amount. On the other hand, for the remaining 5 ligands, the difference was greater than 1 kcal/mol and in one case, for ligand 18, as high as 3.4 kcal/mol. This suggests that these interactions are quite complex and not well modeled by the scoring function. However, part of the reason for the failure of the scoring function might be that the “correct” poses were not perfect; only one of the 5 ligands with large score difference had an RMSD below 1 Å towards experiment. In particular, the best found docking pose of ligand 18 (RMSD 1.2 Å) had one amide group oriented in the wrong direction, thus preventing the formation of a hydrogen bond with His-451; this can probably explain the rather poor score and the resulting prediction of an unrelated pose (RMSD 8.8 Å) as the best one (see Fig. S2 in the Supplementary material).

**Table 2:** Summary of the docking results

Cutoff (Å)	Number of correct poses					
	First	Best	Globally first	Globally best	*ixnzu*	*7itmc*
1.0	7	11	11	22	17	18
1.5	11	19	17	27	23	21
2.0	13	21	19	29	26	26
2.5	13	22	20	30	28	26
3.0	13	24	20	33	29	26

Each line reports the number of ligands for which the various docking strategies in Table 1 found the “correct” pose, if evaluated by a given RMSD cutoff threshold. Just as in Table 1, the “first” pose is the submitted top-scored pose, the “best” pose is the pose that retrospectively was the best of the five submitted poses, the “globally best” is the retrospectively best pose among the dockings to all (including secret) crystal structures, and the “globally first” pose is the top-scored pose among the dockings to the whole set of protein structures. For comparison, the corresponding best-pose results are shown for the two best submissions in the challenge: *ixnzu* and *7itmc*. The 2 Å threshold is the default used throughout this study

**Table 3:** Average RMSD in Å for the various types of ligands using the best docking result from the blind stage (old) and the analysis stage (global), respectively

Group	Old	Global
Benzimidazoles (benz)	1.36	0.71
Sulfonamides (sulf)	2.32	0.87
Isoxazoles (isox)	3.61	1.09
Spirocycles (spir)	4.02	1.90
Miscellaneous (misc)	5.50	2.33

For comparison, the results for the two best submissions in the challenge (*7ltme* and *ixnzu*) have been included in Table 2. When preparing these submissions, the authors used two commercial docking programmes, GLIDE/GOLD and ICM Dock, respectively. Interestingly, previous comparative studies of docking programmes have indicated that both these programmes out-perform Autodock Vina for pose prediction [49, 50], thus the results for the FXR system seem to be consistent with previous results.

### MD refinement

A subset of the docking poses were selected for refinement using classical MD simulations, followed by clustering, as described in the method section. Originally, this subset included the top-predicted pose of each ligand; the cluster centers of these simulations were submitted as a separate entry (*byf51*) in the challenge, but with no improvement relative to the docking results. Later, the set of MD simulations was extended to include all docking poses that were within 3 Å of the experimental structure, regardless of their initial rank. This enabled us to collect more statistics on the possible use of MD for refinement of *reasonably* correct poses, as we did not expect MD to repair the mis-docked ones. In total, 48 simulations starting from docking poses were run (but the one for ligand 33 was discarded). For comparison, a series of simulations starting from the experimentally obtained binding pose for each ligand were also performed (35 additional simulations).

The results of all the simulations are given in Table 4. The MD simulations were remarkably stable; only four of the simulations (for ligands 3, 5, 10, and 16) diverged substantially from the initial pose (according to the definition in the method section). This means that not only were all the simulations starting from a pose close to experiment stable, but also a great majority of the simulations starting from a totally wrong binding pose. Apparently, the docking method did well in predicting poses that were at least kinetically stable, and our simple MD refinement procedure was not able to further pinpoint the poses which are most stable in a thermodynamic sense. Plots of the RMSD along all trajectories are given in Fig. S3 in the Supplementary material.

**Fig. 2:**
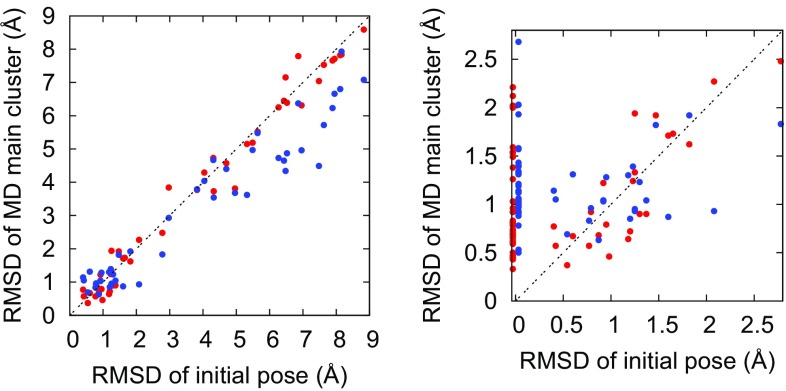
Scatter plot showing the resulting RMSD towards experiment for the main cluster in the MD simulation (red) and the best cluster in the RMD simulation (blue), for a given RMSD of the initial pose. The line represents no change in RMSD. The right plot is merely a magnification showing the range 0–2.8 Å, corresponding to “reasonably good” initial poses. The right plot also includes the simulations started from experimental poses (which all have an initial RMSD of zero but are slightly displaced along the x axis to avoid cluttering)

On average, the RMSD towards experiment changed insignificantly (by 0.06 Å) in the MD refinement, reflecting an almost equal probability of improvement (60%) and deterioration (40%). The individual variation is shown in Fig. 2, where a red circle marks an improvement if it is below the diagonal line. Several ligands whose docking poses had RMSDs in the range 1–1.5 Å were improved to RMSDs below 1 Å, indicating a potential usefulness of the approach, but, as already mentioned, several poses instead became worse. Running multiple simulations from each starting point would obviously have increased the probability of improving each pose, but without a reliable way of picking out the correct candidate from the simulations, such protocol would not have helped in a blind-challenge context.

The results for the simulations that were started from the experimental structures are also given in Table 4. Interestingly, 32 of the ligands gave the correct pose as the main cluster center. In other words, for only 3 of the ligands (10, 11, and 17) the simulation diverged more than 2 Å from the experimental (starting) structure, and none of them more than 3 Å. For one of these (ligand 17), the set of clusters actually included a pose close to the experiment (RMSD 0.6 Å), but it was not the largest cluster and was visited only in the beginning of the simulation. Ligand 11 will be further discussed in a separate section. For some ligands, the MD simulation starting from the experimental pose resulted in a cluster center with slightly higher RMSD towards experiment than the simulation starting from a docking pose. This apparent paradox is most likely an effect of randomness; independent simulations started from both structures would presumably have given a similar range of structures.

**Table 4:** Summary of the MD and RMD simulations. For each ligand, up to three initial poses are considered. For each pose, the table gives the PDB ID of the crystal structure used as starting structure for the protein, the RMSD between the initial pose and experiment (init/exp; same as in Table 1), the RMSD between the MD main cluster and experiment (MD/exp), the RMSD between the MD cluster and the initial pose (MD/init), the minimum RMSD between a RMD cluster and experiment (RMD/exp), the maximum RMSD between a RMD cluster and the initial pose (RMD/init), and the number of clusters obtained in the RMD simulation. All RMSDs towards the experimental structure were calculated by the official script taking into account symmetry-equivalent atoms, whereas the rest of the RMSDs were calculated atom by atom (thus the MD/exp and MD/init values may differ even for the simulations started from experiment). In all cases, the RMSD was calculated for all non-hydrogen atoms of the ligand after aligning the $\hbox {C}_\alpha$ atoms of the protein. All RMSD values are given in Å

Lig	Pose	PDB	init/exp	MD/exp	MD/init	RMD/exp	RMD/init	# Clusters
1	First	3OMK	5.32	5.15	0.76	3.62	5.20	26
	Best	3OLF	2.97	3.84	3.06	2.93	5.37	20
	Exp			0.82	0.76	1.43	2.90	7
2	First	3OMM	6.86	7.79	0.57	6.37	6.30	19
	Exp			0.43	0.45	0.98	7.30	25
3	First	3P88	4.70	4.57	2.38	4.40	7.50	7
	Exp			1.54	1.85	0.91	2.60	4
4	First	3P88	3.82	3.77	1.42	3.79	6.60	30
	Exp			1.26	1.32	1.40	6.00	15
5	First	3OMM	6.48	7.15	3.09	4.34	7.40	13
	Exp			0.33	0.33	0.53	5.30	5
6	First	3OMK	0.42	0.57	0.54	1.05	6.50	15
	Exp			0.65	0.60	1.05	5.40	9
7	First	3OMM	1.20	0.72	0.71	0.85	5.80	16
	Exp			0.83	1.34	1.31	5.30	9
8	First	3OMM	5.49	5.19	1.12	4.97	5.50	14
	Best	3OMM	0.92	1.04	1.02	1.03	5.02	5
	Exp			0.92	1.44	0.98	8.60	13
9	First	3OMK	0.54	0.37	0.49	0.69	4.70	12
	Exp			0.46	0.97	1.03	8.40	21
10	First	1OSH	4.96	3.81	1.18	3.68	13.30	32
	Exp			2.02	1.81	2.68	5.60	10
11	First	1OSH	4.32	3.73	1.62	3.54	10.70	33
	Exp			2.12	2.16	1.93	6.80	16
12	First	1OSH	4.31	4.73	2.47	4.67	12.90	28
	Best	3FLI	2.78	2.48	1.56	1.83	4.58	15
	Exp			2.00	2.68	2.03	4.50	14
13	First	3OLF	7.95	7.71	1.28	6.66	6.20	18
	Exp			0.50	0.48	1.34	7.10	9
14	First	3OLF	0.87	0.68	1.32	0.63	6.90	25
	Exp			0.72	0.63	0.88	7.60	13
15	$\hbox {First}^*$	3OMM	7.48	7.04	0.71	4.49	16.80	49
	Exp			1.49	1.76	1.57	4.30	10
16	First	3DCU	8.16	7.84	1.59	7.93	9.20	18
	Best	3FLI	1.25	1.94	1.62	0.95	4.33	10
	Exp			1.53	1.76	1.58	6.30	13
17	First	3FLI	1.47	1.92	1.85	1.82	3.70	10
	Exp			2.21	2.25	2.03	5.40	14
18	First	3OOF	8.83	8.59	1.43	7.08	8.10	20
	Exp			0.92	0.88	0.93	8.80	19
19	First	3OMM	1.30	0.90	0.70	1.23	7.60	26
	Exp			0.96	1.29	1.01	6.10	7
20	First	3OMM	6.96	6.31	1.05	4.96	7.60	24
	Best	3OMK	0.60	0.67	0.55	1.31	5.42	11
	Exp			0.70	1.01	0.97	8.60	18
21	First	3OMK	6.27	6.25	0.56	4.73	5.90	16
	Best	3OMM	0.95	0.79	1.15	1.28	5.85	8
	Exp			0.67	1.11	1.13	7.90	18
22	First	3OMM	0.92	1.22	0.66	1.04	5.20	13
	Exp			1.38	1.64	1.14	5.00	13
23	First	3HC6	7.63	7.53	0.66	5.72	8.60	39
	Exp			1.03	0.89	1.21	8.10	25
24	First	3OMK	6.52	6.39	0.75	4.87	6.10	13
	Best	3OMM	1.18	0.64	1.00	1.30	5.93	9
	Exp			0.69	1.07	1.33	7.40	11
25	First	3OMM	1.65	1.73	0.68	$\hbox {N/A}^\#$	$\hbox {N/A}^\#$	$\hbox {N/A}^\#$
	Best	3OMM	0.98	0.46	1.35	$\hbox {N/A}^\#$	$\hbox {N/A}^\#$	$\hbox {N/A}^\#$
	Exp			0.47	0.60	$\hbox {N/A}^\#$	$\hbox {N/A}^\#$	$\hbox {N/A}^\#$
26	First	3OMK	7.89	7.66	1.48	6.23	4.90	8
	Best	3OMM	1.23	1.24	1.10	1.39	5.67	5
	Exp			0.81	0.89	0.99	4.90	6
27	First	3OMM	1.25	1.33	0.81	0.93	6.00	15
	Exp			0.74	1.20	0.50	5.20	8
28	First	3OMM	0.77	0.57	0.92	0.83	6.00	17
	Exp			0.62	1.05	1.02	7.30	13
29	First	3OMM	0.79	0.92	0.71	0.96	7.00	15
	Exp			0.60	1.01	0.91	4.30	7
30	First	3OMK	6.43	6.45	0.78	4.65	4.30	12
	Best	3OLF	1.82	1.62	1.02	1.92	5.70	6
	Exp			0.78	1.03	0.78	5.70	9
31	First	3OMM	1.60	1.71	0.60	0.87	2.30	3
	Exp			0.59	0.99	1.33	5.10	12
32	First	3OMM	4.04	4.29	1.32	4.04	3.30	9
	Best	3OMM	2.08	2.27	1.25	0.93	4.33	11
	Exp			0.80	1.16	1.12	5.10	16
34	$\hbox {First}^*$	3OLF	5.64	5.52	1.47	5.48	12.60	15
	Exp			1.59	1.56	1.39	7.50	12
35	First	3OOF	0.40	0.77	1.48	1.14	1.20	1
	Exp			0.44	0.90	1.19	1.40	1
36	First	3OMM	8.12	7.82	1.24	6.80	6.40	13
	Best	3OMM	1.37	0.90	1.16	1.04	5.81	3
	Exp			0.92	0.93	1.06	6.30	5

$^*$The simulation was started from another docking pose than that given in Table 1, but with similar score

$^\#$These simulations crashed, probably due to the RMD bias pushing some dihedral angles into ranges where the force field is numerically unstable when used with GROMACS 4.6.2

The reason for the experiment-based simulation poses deviating from the experiment for some ligands is probably the limited accuracy of the force field, which is caused by the use of generic torsional parameters as well as the intentionally “blind” preparation of partial charges for each ligand (i.e. not exploiting any knowledge of the experimental pose).

**Fig. 3:**
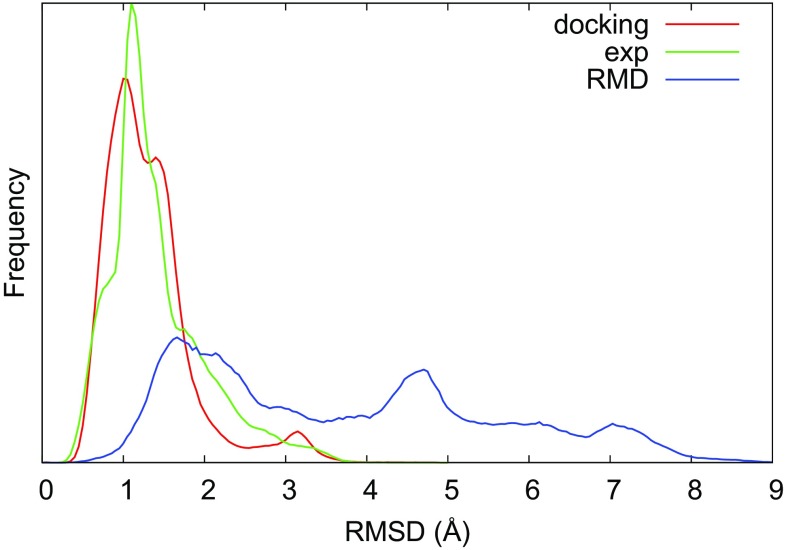
Distribution of RMSD from the initial structure over all MD simulations started from docking poses (dock) or experimental structures (exp), as well as all RMD simulations started from the experimental structures (RMD)

On the other hand, the reason for most simulations staying close to the starting structure despite force field deficiencies is most likely the relatively short simulation times, which did not allow for larger rotation of the ligand in the binding site. Indeed, both the docking-based and experiment-based simulations showed a similar distribution of the RMSD during the simulations (see Fig. 3), and a similar average RMSD between the main cluster center and the initial structure (1.20 Å for docking-based, 1.19 Å for experiment-based).

Finally, we tested whether the MD trajectories could instead be used in a MM-PBSA context to help ranking the binding poses. More specifically, we selected six example ligands, for which the Autodock Vina scoring function mis-ranked the poses. For these ligands, we investigated whether MM-PBSA provided sufficient precision to discriminate between various binding poses and, in such case, whether it ranked the poses in agreement with their RMSD towards experiment. The details of this investigation, including a thorough discussion of the results, are given in the section *MM-PBSA analysis of MD trajectories* in the Supplementary material. In summary, the method correctly predicted the experimental pose to have the most negative binding free energy for all six tested ligands (see Table S7 in the Supplementary material) and thus seems to be useful for this particular purpose. However, especially for the charged ligands, it was difficult to estimate the systematic error in applying the method to different poses, and thus further investigation would be needed to establish the significance of the results [51].

### Reconnaissance metadynamics simulations

To investigate whether reconnaissance metadynamics (RMD) can be used to enhance the sampling and thereby explore new binding poses, we performed RMD simulations using the dihedral angles of the rotable bonds in each ligand as collective variables (CVs). During the blind challenge, only three systems (ligands 22, 27, and 32) were subjected to RMD simulations due to the limited time, and no improvement was obtained for these (in fact two of them turned out to be already in the correct binding pose). Later, we extended the RMD investigation to include all the MD-simulated systems (i.e. those started from docking poses as well as from experimental poses) to obtain better statistics.

The results of the RMD simulations are summarized in Table 4 next to the corresponding MD simulations. To mimic the typical blind usage of the method, only the set of significant cluster centers (as defined in the method section) were evaluated, i.e. not the full set of simulation frames. From the set of cluster centers, the table reports the minimum RMSD towards the experimental structure, as well as the maximum RMSD towards the starting structure. To enable fair comparisons with the MD results, the starting structure of the MD simulation was used as the reference in both cases, and the RMSD was computed “atom by atom” without taking account of symmetry.

**Fig. 4:**
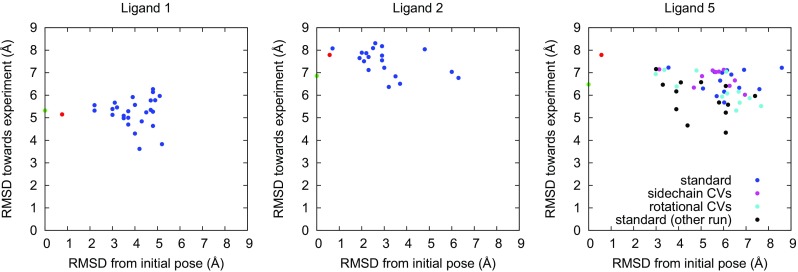
Example of the behavior of RMD for the arbitrary ligands 1 and 2, and for the various choices of CVs for ligand 5. The scatter plots show the RMSD towards the initial pose versus the RMSD towards experiment for each of the RMD clusters (blue), as well as for the MD cluster (red) and the initial docking pose (green), for reference. For ligand 5, results for three RMD variants are shown: the standard settings with only ligand dihedrals (blue), the inclusion of sidechain CVs (magenta), and the inclusion of rotation-promoting CVs (cyan). In addition, the results for an independent simulation with the standard settings are shown (black); the latter is used for the analysis in Table 4. Note that the simulation with rotational CVs was only run for 8 ns due to technical problems; significantly more clusters would probably have been visited if it had been run for 20 ns like all the others

On average, a minor improvement in the RMSD towards experiments was obtained (by 0.6 Å). More importantly, the RMSD of the “farthest” RMD cluster was significantly higher than that of the MD cluster for all ligands (the averages being 6.1 and 2.5 Å, respectively). From the overall distribution of the RMSD shown in Fig. 3, it can be seen that the difference is not caused by a single pose with high RMSD, but the whole RMSD distribution is shifted; clearly, the RMD simulations explore binding poses much farther from the initial pose than MD simulations of a corresponding length. Two examples of the typical exploration are shown in Fig. 4. As can be seen, the far-lying poses can be either closer to or farther from the experimental pose, a seemingly random behavior. For almost all ligands, a significant number of distinct poses are explored (see Table 4). For one ligand (35), only one cluster was obtained and its RMSD towards the starting structure was within 1.5 Å, regardless of whether starting from the docking pose or the experimental structure). Visual inspection of the RMD trajectory revealed that, although many configurations of the seven rotable bonds were explored, the chemical groups of the ligand were held in place by on average $\sim$ 5 hydrogen bonds (see Fig. S4 in the Supplementary material), thus causing the ligand to “wriggle” at its position instead of exploring fundamentally new binding poses.

For three ligands (5, 13, and 15), we performed additional RMD simulations with an extended set of CVs including seven dihedral angles of protein sidechains that were identified by visual inspection as possibly restricting the ligand movement. The purpose of these simulations was to see whether the exploration of new poses would be stimulated if the enhanced sampling of the ligand dihedrals was accompanied by enhanced sampling of these selected sidechain dihedrals. For example, one could imagine that a certain rotation of the ligand could, due to steric hindrance, only take place when a certain sidechain adopts a particular conformation. Covariance between the two types of fluctuations could then in principle be detected by the RMD algorithm, with the applied bias increasing the probability of the two movements to occur simultaneously.

Interestingly, the introduction of sidechain CVs actually decreased the fluctuation of the ligand (see Fig. S5 in the Supplementary material). The reason for this counterintuitive behavior is probably that RMD is designed to find the path with the lowest energy barrier out of a given basin. If the sidechain CVs are “softer” degrees of freedom with lower energy barriers between the local minima than the ligand dihedrals, the algorithm will seek the “easy way out” and predominantly enhance the fluctuations of the sidechain CVs, thus exploring the conformational space of the ligand at a lower pace than without the sidechain CVs. Indeed, the obtained poses were less varied in the simulation with sidechain CVs, as demonstrated in Fig. S6, S7 in the Supplementary material.

Finally, for one ligand (5), we tested to include three CVs related to the orientation of the ligand relative to the protein (see Fig. S1 in the Supplementary material), in addition to the ligand dihedral CVs (but no sidechain CVs). The purpose of the orientational CVs was to enhance the rotational movement of the ligand, thereby counteracting the steric restrictions imposed by the surrounding amino acids. The results show that the ligand rotated much more when these CVs were included (see Fig. S5 in the Supplementary material). Moreover, the RMSD towards the starting structure grew faster and the number of explored poses increased, as illustrated in Fig. 4, although it should be emphasized that the variation among individual runs was large, as can be seen for the two equivalent runs with standard settings. A visual inspection of the explored poses also confirmed that the poses became more diverse when rotational CVs were included (see Fig. S8 in the Supplementary material). Unfortunately, this simulation became unstable and crashed frequently, probably because the biasing forces pushed the ligand into regions of the conformational space in which the rings of the molecule were strained and the force field parameters inadequate. Still, the preliminary results are promising and we intend to explore this possibility further in the future.

### Binding pose analysis of a typical ligand

**Fig. 5:**
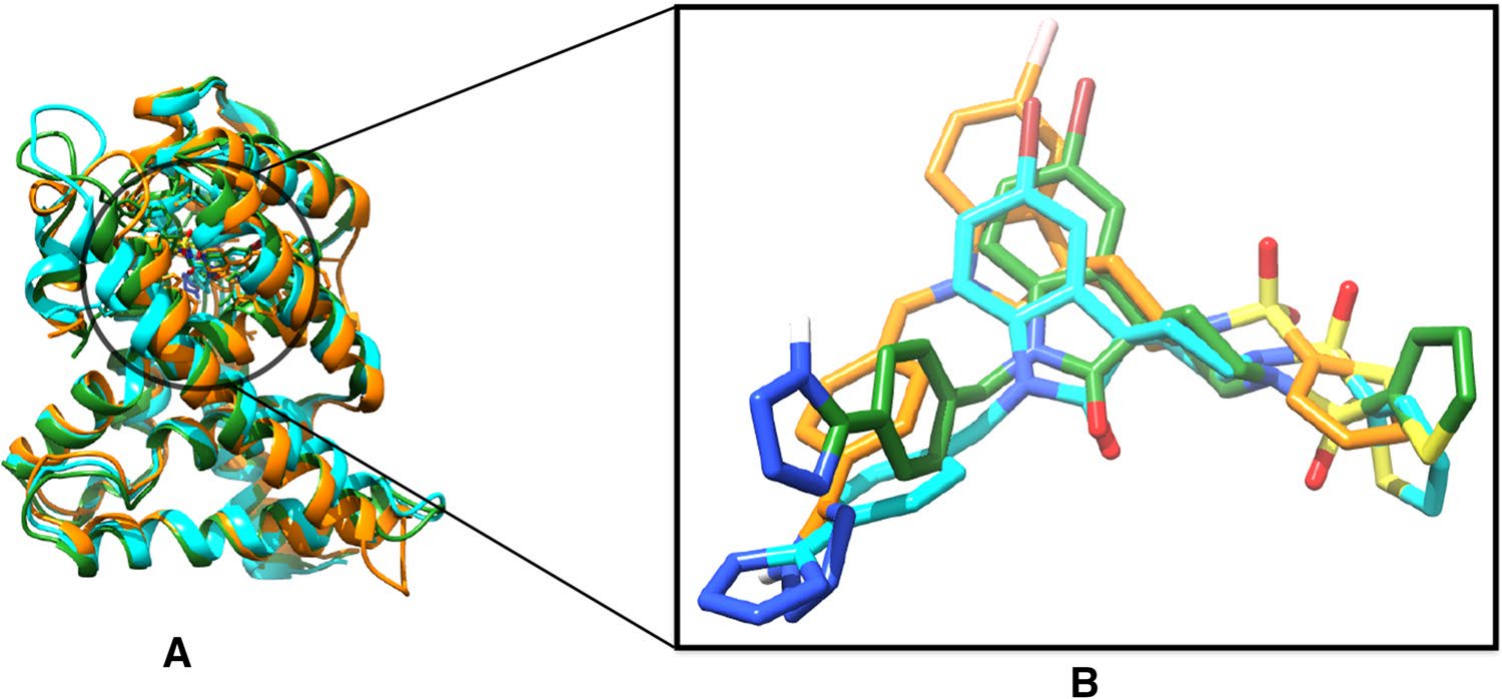
Protein–ligand complex for ligand 11 from experiment (cyan), MD simulation (green) and RMD simulation (orange) superimposed over each other (**a**). The magnification shows only the ligand of these three complexes and highlights the difference in binding pose among them (**b**)

Ligand 11 was selected for a more detailed investigation because it diverged more than 2 Å during the MD simulation starting from the experimental crystal structure. The best cluster of the RMD simulation had slightly smaller RMSD (1.9 Å), so it is interesting to investigate not only how the simulated poses differed from the experimental pose in terms of interactions, but also what caused the slight improvement in the RMD pose. From a visual inspection (see Fig. 5b), it is evident that the complex obtained from MD simulation had a flipped tetrazole group compared to the experimental structure. In the RMD pose, on the other hand, the tetrazole group adopted a similar orientation as in the experiment despite differences in the neighboring parts of the molecule. In both the MD and RMD poses, the terminal thiophene ring was arranged slightly differently compared to experiment, thus contributing to the high RMSD.

**Fig. 6:**
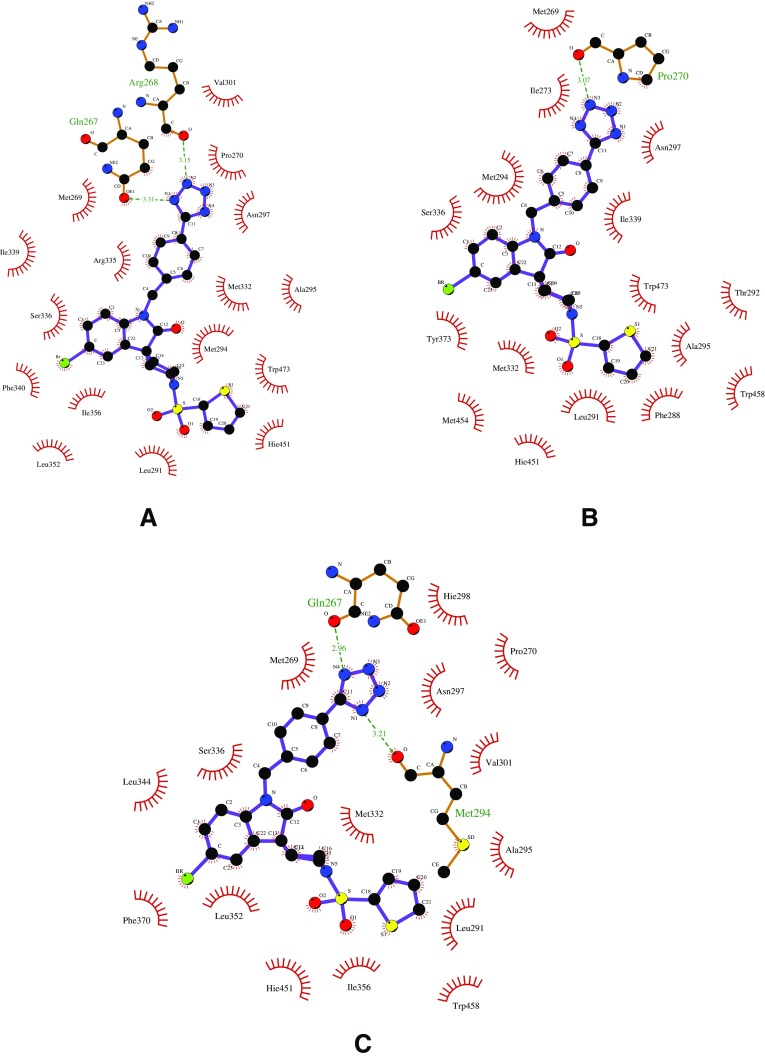
2D ligand interaction map of complexes for ligand 11 from experiment (**a**), MD simulation (**b**) and RMD simulation (**c**). Hydrogen bonds are depicted as* dashed lines* between the atoms involved, whereas hydrophobic contacts are represented by an arc with spokes radiating towards the ligand atom. Protein–ligand interaction analysis was performed using UCSF Chimera and LigPlot [52] software. LigPlot automatically generates schematic diagrams for protein–ligand interaction for a given geometry

A protein–ligand interaction analysis for these three complexes further highlighted these differences. In the experimental structure, the tetrazole ring of the ligand formed two hydrogen bond interactions with Gln-267 and Arg-268, respectively (Fig. 6a). However, in the MD pose, due to the flipped orientation of the tetrazole ring, it did not form any hydrogen bond interactions with Gln-267 or Arg-268; instead it formed a new hydrogen bond with a neighbouring residue, Pro-270 (Fig. 6b). During the enhanced sampling in the RMD simulation, the ligand restored the hydrogen bond interaction with Gln-267, but instead of Arg-268 it formed a new hydrogen bond with Met-294 (Fig. 6c). Some changes in the interaction pattern were also seen around the thiophene ring due to its different orientation.

Visual inspection of the MD trajectory showed that the tetrazole ring “wiggled” for a very short period of time and visited a “flipped” conformation but mostly remained in the same conformation, that of Fig. 6b. In the RMD simulation, the thiophene and the tetrazole rings visited different conformations due to the bias applied to the rotable bonds, but mostly remained close to the conformation of Fig. 6c, which was stabilised by hydrogen bonds and hydrophobic interactions.

## Conclusions

Our stepwise approach allowed us to draw several conclusions on the performance of molecular docking, molecular dynamics (MD) and reconnaissance metadynamics (RMD) when used as pose-prediction methods for this particular system.

The procedure to use multiple protein structures for docking to account for the known conformational variation worked very well in this case, allowing us to find correct binding poses for 21 of the 35 ligands and placing our docking submission in the upper half of the submissions to the D3R Grand challenge 2. However, for 8 of these successful ligands, the correct pose was not predicted as the top pose, which suggests room for improvement in the scoring function used in Autodock Vina. Inclusion of the new (secret) protein structures in the docking set would have increased the number of correct poses to 29, but still 6 ligands would have remained mis-docked, most of them classified as miscellaneous. This confirms the data set as rather difficult.

The MD refinement of the docking poses did not provide any significant improvement; in fact the RMSD towards experiment increased in almost half of the cases. The simulations showed a remarkable kinetic stability for almost all of the docking poses, regardless whether they were correct or not. Thus, stability in MD simulations of length $\sim$ 30 ns can not be used as a discriminator for the correctness of binding poses. A complementary set of MD simulations, started directly from the experimental binding poses, revealed some force field deficiencies, but overall the automatic GAFF parametrization seemed to work well for these ligands and most of them remained close to the experimental structure.

The subsequent RMD simulations were successful in exploring new binding poses, but never visited the experimental structure if started from a mis-docked pose. One of the problems seems to be that by only applying bias potentials on the internal dihedrals of the ligand, one does not promote rotation of the ligand with respect to the protein. Another problem is that the tight environment around the binding site sterically restricts the ligand’s exploration of conformational states. Preliminary tests were made to include collective variables that tackle each of these problems, but further development is needed to accomplish this task. In the future, we believe that such carefully devised collective variables may contribute towards a reliable method for improving docking poses by simulations. An important problem that remains, however, is how to reliably identify the most stable pose from trajectories of a limited length.

## Electronic supplementary material

Below is the link to the electronic supplementary material.

Supplementary material 1 (PDF 11526 kb)
