# Supplementary material for: Prediction of binding poses to FXR using multi-targeted docking combined with molecular dynamics and enhanced sampling
Source: J Comput Aided Mol Des. 2017 Oct 20;32(1):59–73. doi: 10.1007/s10822-017-0074-x (PMC5767213; doi:10.1007/s10822-017-0074-x)
Supplement: Supplementary file 1 — Supplementary material 1 (PDF 11526 kb) [file 10822_2017_74_MOESM1_ESM.pdf]

Supplementary material

S. Bhakat, E. Åberg, P. Söderhjelm

Prediction of binding poses to FXR using multi-targeted docking combined with molecular dynamics and enhanced sampling

Table S1 The PDB IDs for the complexes used to extract protein coordinates for the molecular docking.

1OSH
3FLI
3FXV
3L1B
3OKH
3OLF
3OMK
3OMM
3OOF
4QE6
4QE8
3DCT
3DCU
3HC5
3HC6
3P88
3RUT
3RUU

Table S2 List of ligands treated by RMD in the RMD submission to the challenge, and the number of clusters considered for each ligand. More specifically, the submitted poses were obtained in the following way: First, the MD and RMD trajectories were merged and clustered together. To confirm the stability of the resulting poses, the cluster centers of the top 4–5 clusters were used as starting points for additional MD simulations, each of length 20 ns (except the MD pose itself, for which the previous MD simulation was simply adopted). Finally, each MD simulation was clustered separately and the top cluster center was used as the submitted pose in the RMD submission. The ranking of the poses was determined by a manual procedure taking into account the size of the clusters in the combined clustering, the stability of the pose in the extra MD simulation, and the *ad hoc* decision not to rank the MD pose as the top pose.

Ligand	Number of clusters
22	4
27	5
32	5

Table S3 Amino-acid sequences used for the various simulations (table extending into the next four pages). The secret crystal structures had greater variation in the sequence, sometimes involving insertions and deletions that could not be easily restored without affecting the geometry. We opted for a compromise: Extra terminal residues (if present) were deleted but other insertions and deletions were kept as in the crystal structures. Point mutations anywhere in the structure were put back according to the apo-protein using the graphical user interface of UCSF Chimera and the `swapaa` command. The *Dock Prep* module of UCSF Chimera was used to keep only the highest occupancy for atoms with alternative positions, and was also used to replace each truncated side-chains with a complete side-chain of the same residue type [35]. The "gaps" in the backbone (poorly resolved residues that were missing in the PDB files) were filled by adding the missing residues from the apo-template and optimizing the added sequence using the ModLoop [36] web server, which optimizes loop conformations without relying on known protein structures. The final amino acid sequence of each system is given below. In analogy with the procedure for the old crystal structures, the protonation state of the new structures were set identical to the apo structure.

Structure	Sequence
**Apo**	MELTPDQQTLLHFIMDSYNKQRMPQEITNKILKEAFSAEENFLILTEMATNHVQVLVEFT<br>KKLPGFQTLDHEDQIALLKGSAVEAMFLRSAEIFNKKLPAGHSDLLEARIRNSGISDEYI<br>TPMFSFYKSIGELKMTQEEYALLTAIVILSPDRQYIKDREAVEKLQEPLLDVLQKLCKIHQ<br>PENPQHFACLLGRLTELRTFNHHHAEMLMSWAKFTPLLCEIWD
**Our submissions**	ELTPDQQTLLHFIMDSYNKQRMPQEITNKILKEAFSAEENFLILTEMATNHVQVLVEFTK<br>KLPGFQTLDHEDQIALLKGSAVEAMFLRSAEIFNKKLPAGHSDLLEARIRNSGISDEYIT<br>PMFSFYKSIGELKMTQEEYALLTAIVILSPDRQYIKDREAVEKLQEPLLDVLQKLCKIHQP<br>ENPQHFACLLGRLTELRTFNHHHAEMLMSWAVNDHKFTPLLCEIWD
**Secret Structures'**	
**FXR-Lig1**	ELTPDQQTLLHFIMDSYNKQRMPQEITNKILKEAFSAEENFLILTEMATNHVQVLVEFTK<br>KLPGFQTLDHEDQIALLKGSAVEAMFLRSAEIFNKKLPAGHSDLLEARIRNSGISDEYIT<br>PMFSFYKSIGELKMTQEEYALLTAIVILSPDRQYIKDREAVEKLQEPLLDVLQKLCKIHQP<br>ENPQHFACLLGRLTELRTFNHHHAEMLMSWAVNDHKFTPLLCEIWD
**FXR-Lig2**	ELTPDQQTLLHFIMDSYNKQRMPQEITNKILKEAFSAEENFLILTEMATNHVQVLVEFTK<br>KLPGFQTLDHEDQIALLKGSAVEAMFLRSAEIFNKKLPAGHSDLLEARIRNSGISDEYIT<br>PMFSFYKSIGELKMTQEEYALLTAIVILSPDRQYIKDREAVEKLQEPLLDVLQKLCKIHQP<br>ENPQHFACLLGRLTELRTFNHHHAEMLMSWAVNDHKFTPLLCEIWD
**FXR-Lig3**	ELTPDQQTLLHFIMDSYNKQRMPQEITNKILKEAFSAEENFLILTEMATNHVQVLVEFTK<br>KLPGFQTLDHEDQIALLKGSAVEAMFLRSAEIFNKKLPAGHSDLLEARIRNSGISDEYIT<br>PMFSFYKSIGELKMTQEEYALLTAIVILSPDRQYIKDREAVEKLQEPLLDVLQKLCKIHQP<br>ENPQHFACLLGRLTELRTFNHHHAEMLMSWAVNDHKFTPLLCEIWD
**FXR-Lig4**	ELTPDQQTLLHFIMDSYNKQRMPQEITNKILKEAFSAEENFLILTEMATNHVQVLVEFTK<br>KLPGFQTLDHEDQIALLKGSAVEAMFLRSAEIFNKKLPAGHSDLLEARIRNSGISDEYIT<br>PMFSFYKSIGELKMTQEEYALLTAIVILSPDRQYIKDREAVEKLQEPLLDVLQKLCKIHQP<br>ENPQHFACLLGRLTELRTFNHHHAEMLMSWAVNDHKFTPLLCEIW
**FXR-Lig5**	ELTPDQQTLLHFIMDSYNKQRMPQEITNKILKEAFSAEENFLILTEMATNHVQVLVEFTK<br>KLPGFQTLDHEDQIALLKGSAVEAMFLRSAEIFNKKLPAGHSDLLEARIRNSGISDEYIT<br>PMFSFYKSIGELKMTQEEYALLTAIVILSPDRQYIKDREAVEKLQEPLLDVLQKLCKIHQP<br>ENPQHFACLLGRLTELRTFNHHHAEMLMSWAVNDHKFTPLLCEIWD
**FXR-Lig5**	ELTPDQQTLLHFIMDSYNKQRMPQEITNKILKEAFSAEENFLILTEMATNHVQVLVEFTK<br>KLPGFQTLDHEDQIALLKGSAVEAMFLRSAEIFNKKLPAGHSDLLEARIRNSGISDEYIT<br>PMFSFYKSIGELKMTQEEYALLTAIVILSPDRQYIKDREAVEKLQEPLLDVLQKLCKIHQP<br>ENPQHFACLLGRLTELRTFNHHHAEMLMSWAVNDHKFTPLLCEIWD
**FXR-Lig6**	ELTPDQQTLLHFIMDSYNKQRMPQEITNKILKEAFSAEENFLILTEMATNHVQVLVEFTK<br>KLPGFQTLDHEDQIALLKGSAVEAMFLRSAEIFNKKLPAGHSDLLEARIRNSGISDEYIT<br>PMFSFYKSIGELKMTQEEYALLTAIVILSPDRQYIKDREAVEKLQEPLLDVLQKLCKIHQP<br>ENPQHFACLLGRLTELRTFNHHHAEMLMSWAVNDHKFTPLLCEIWD
**FXR-Lig7**	ELTPDQQTLLHFIMDSYNKQRMPQEITNKILKEAFSAEENFLILTEMATNHVQVLVEFTK<br>KLPGFQTLDHEDQIALLKGSAVEAMFLRSAEIFNKKLPAGHSDLLEARIRNSGISDEYIT<br>PMFSFYKSIGELKMTQEEYALLTAIVILSPDRQYIKDREAVEKLQEPLLDVLQKLCKIHQP<br>ENPQHFACLLGRLTELRTFNHHHAEMLMSWAVNDHKFTPLLCEIWD
**FXR-Lig8**	ELTPDQQTLLHFIMDSYNKQRMPQEITNKILKEAFSAEENFLILTEMATNHVQVLVEFTK<br>KLPGFQTLDHEDQIALLKGSAVEAMFLRSAEIFNKKLPAGHSDLLEARIRNSGISDEYIT<br>PMFSFYKSIGELKMTQEEYALLTAIVILSPDRQYIKDREAVEKLQEPLLDVLQKLCKIHQP<br>ENPQHFACLLGRLTELRTFNHHHAEMLMSWAVNDHKFTPLLCEIWD

Structure	Sequence
**FXR-Lig9**	ELTPDQQTLLHFIMDSYNKQRMPQEITNKILKEAFSAEENFLILTEMATNHVQVLVEFTK<br>KLPGFQTLDHEDQIALLKGSAVEAMFLRSAEIFNKKLPAGHSDLLEARIRNSGISDEYIT<br>PMFSFYKSIGELKMTQEEYALLTAIVILSPDRQYIKDREAVEKLQEPLLDVLQKLCKIHQP<br>ENPQHFACLLGRLTELRTFNHHHAEMLMSWAVNDHKFTPLLCEIWD
**FXR-Lig10**	ELTPDQQTLLHFIMDSYNKQRMPQEITNKILKEAFSAEENFLILTEMATNHVQVLVEFTK<br>KLPGFQTLDHEDQIALLKGSAVEAMFLRSAEIFNKKLPAGHSDLLEARIRNSGISDEYIT<br>PMFSFYKSIGELKMTQEEYALLTAIVILSPDRQYIKDREAVEKLQEPLLDVLQKLCKIHQP<br>ENPQHFACLLGRLTELRTFNHHHAEMLMSWAVNDHKFTPLLCEIWD
**FXR-Lig11**	ELTPDQQTLLHFIMDSYNKQRMPQEITNKILKEAFSAEENFLILTEMATNHVQVLVEFTK<br>KLPGFQTLDHEDQIALLKGSAVEAMFLRSAEIFNKKLPAGHSDLLEARIRNSGISDEYIT<br>PMFSFYKSIGELKMTQEEYALLTAIVILSPDRQYIKDREAVEKLQEPLLDVLQKLCKIHQP<br>ENPQHFACLLGRLTELRTFNHHHAEMLMSWAVNDHKFTPLLCEIWD
**FXR-Lig12**	ELTPDQQTLLHFIMDSYNKQRMPQEITNKILKEAFSAEENFLILTEMATNHVQVLVEFTK<br>KLPGFQTLDHEDQIALLKGSAVEAMFLRSAEIFNKKLPAGHSDLLEARIRNSGISDEYIT<br>PMFSFYKSIGELKMTQEEYALLTAIVILSPDRQYIKDREAVEKLQEPLLDVLQKLCKIHQP<br>ENPQHFACLLGRLTELRTFNHHHAEMLMSWAVNDHKFTPLLCEIWD
**FXR-Lig13**	ELTPDQQTLLHFIMDSYNKQRMPQEITNKILKEAFSAEENFLILTEMATNHVQVLVEFTK<br>KLPGFQTLDHEDQIALLKGSAVEAMFLRSAEIFNKKLPAGHSDLLEARIRNSGISDEYIT<br>PMFSFYKSIGELKMTQEEYALLTAIVILSPDRQYIKDREAVEKLQEPLLDVLQKLCKIHQP<br>ENPQHFACLLGRLTELRTFNHHHAEMLMSWAVNDHKFTPLLCEIWD
**FXR-Lig14**	ELTPDQQTLLHFIMDSYNKQRMPQEITNKILKEAFSAEENFLILTEMATNHVQVLVEFTK<br>KLPGFQTLDHEDQIALLKGSAVEAMFLRSAEIFNKKLPAGHSDLLEARIRNSGISDEYIT<br>PMFSFYKSIGELKMTQEEYALLTAIVILSPDRQYIKDREAVEKLQEPLLDVLQKLCKIHQP<br>ENPQHFACLLGRLTELRTFNHHHAEMLMSWAVNDHKFTPLLCEIWD
**FXR-Lig15**	ELTPDQQTLLHFIMDSYNKQRMPQEITNKILKEAFSAEENFLILTEMATNHVQVLVEFTK<br>KLPGFQTLDHEDQIALLKGSAVEAMFLRSAEIFNKKLPAGHSDLLEARIRNSGISDEYIT<br>PMFSFYKSIGELKMTQEEYALLTAIVILSPDRQYIKDREAVEKLQEPLLDVLQKLCKIHQP<br>ENPQHFACLLGRLTELRTFNHHHAEMLMSWAVNDHKFTPLLCEIWD
**FXR-Lig16**	ELTPDQQTLLHFIMDSYNKQRMPQEITNKILKEAFSAEENFLILTEMATNHVQVLVEFTK<br>KLPGFQTLDHEDQIALLKGSAVEAMFLRSAEIFNKKLPAGHSDLLEARIRNSGISDEYIT<br>PMFSFYKSIGELKMTQEEYALLTAIVILSPDRQYIKDREAVEKLQEPLLDVLQKLCKIHQP<br>ENPQHFACLLGRLTELRTFNHHHAEMLMSWAVNDHKFTPLLCEIWD
**FXR-Lig17**	ELTPDQQTLLHFIMDSYNKQRMPQEITNKILKEAFSAEENFLILTEMATNHVQVLVEFTK<br>KLPGFQTLDHEDQIALLKGSAVEAMFLRSAEIFNKKLPAGHSDLLEARIRNSGISDEYIT<br>PMFSFYKSIGELKMTQEEYALLTAIVILSPDRQYIKDREAVEKLQEPLLDVLQKLCKIHQP<br>ENPQHFACLLGRLTELRTFNHHHAEMLMSWAVNDHKFTPLLCEIWD
**FXR-Lig18**	ELTPDQQTLLHFIMDSYNKQRMPQEITNKILKEAFSAEENFLILTEMATNHVQVLVEFTK<br>KLPGFQTLDHEDQIALLKGSAVEAMFLRSAEIFNKKLPAGHSDLLEARIRNSGISDEYIT<br>PMFSFYKSIGELKMTQEEYALLTAIVILSPDRQYIKDREAVEKLQEPLLDVLQKLCKIHQP<br>ENPQHFACLLGRLTELRTFNHHHAEMLMSWAVNDHKFTPLLCEIW
**FXR-Lig19**	ELTPDQQTLLHFIMDSYNKQRMPQEITNKILKEAFSAEENFLILTEMATNHVQVLVEFTK<br>KLPGFQTLDHEDQIALLKGSAVEAMFLRSAEIFNKKLPAGHSDLLEARIRNSGISDEYIT<br>PMFSFYKSIGELKMTQEEYALLTAIVILSPDRQYIKDREAVEKLQEPLLDVLQKLCKIHQP<br>ENPQHFACLLGRLTELRTFNHHHAEMLMSWAVNDHKFTPLLCEIWD
**FXR-Lig20**	ELTPDQQTLLHFIMDSYNKQRMPQEITNKILKEAFSAEENFLILTEMATNHVQVLVEFTK<br>KLPGFQTLDHEDQIALLKGSAVEAMFLRSAEIFNKKLPAGHSDLLEARIRNSGISDEYIT<br>PMFSFYKSIGELKMTQEEYALLTAIVILSPDRQYIKDREAVEKLQEPLLDVLQKLCKIHQP<br>ENPQHFACLLGRLTELRTFNHHHAEMLMSWAVNDHKFTPLLCEIWD

Structure	Sequence
**FXR-Lig21**	ELTPDQQTLLHFIMDSYNKQRMPQEITNKILKEAFSAEENFLILTEMATNHVQVLVEFTK<br>KLPGFQTLDHEDQIALLKGSAVEAMFLRSAEIFNKKLPAGHSDLLEARIRNSGISDEYIT<br>PMFSFYKSIGELKMTQEEYALLTAIVILSPDRQYIKDREAVEKLQEPLLDVLQKLCKIHQP<br>ENPQHFACLLGRLTELRTFNHHHAEMLMSWAVNDHKFTPLLCEIWD
**FXR-Lig22**	ELTPDQQTLLHFIMDSYNKQRMPQEITNKILKEAFSAEENFLILTEMATNHVQVLVEFTK<br>KLPGFQTLDHEDQIALLKGSAVEAMFLRSAEIFNKKLPAGHSDLLEARIRNSGISDEYIT<br>PMFSFYKSIGELKMTQEEYALLTAIVILSPDRQYIKDREAVEKLQEPLLDVLQKLCKIHQP<br>ENPQHFACLLGRLTELRTFNHHHAEMLMSWAVNDHKFTPLLCEIWD
**FXR-Lig23**	ELTPDQQTLLHFIMDSYNKQRMPQEITNKILKEAFSAEENFLILTEMATNHVQVLVEFTK<br>KLPGFQTLDHEDQIALLKGSAVEAMFLRSAEIFNKKLPAGHSDLLEARIRNSGISDEYIT<br>PMFSFYKSIGELKMTQEEYALLTAIVILSPDRQYIKDREAVEKLQEPLLDVLQKLCKIHQP<br>ENPQHFACLLGRLTELRTFNHHHAEMLMSWAVNDHKFTPLLCEIWD
**FXR-Lig24**	ELTPDQQTLLHFIMDSYNKQRMPQEITNKILKEAFSAEENFLILTEMATNHVQVLVEFTK<br>KLPGFQTLDHEDQIALLKGSAVEAMFLRSAEIFNKKLPAGHSDLLEARIRNSGISDEYIT<br>PMFSFYKSIGELKMTQEEYALLTAIVILSPDRQYIKDREAVEKLQEPLLDVLQKLCKIHQP<br>ENPQHFACLLGRLTELRTFNHHHAEMLMSWAVNDHKFTPLLCEIWD
**FXR-Lig25**	ELTPDQQTLLHFIMDSYNKQRMPQEITNKILKEAFSAEENFLILTEMATNHVQVLVEFTK<br>KLPGFQTLDHEDQIALLKGSAVEAMFLRSAEIFNKKLPAGHSDLLEARIRNSGISDEYIT<br>PMFSFYKSIGELKMTQEEYALLTAIVILSPDRQYIKDREAVEKLQEPLLDVLQKLCKIHQP<br>ENPQHFACLLGRLTELRTFNHHHAEMLMSWAVNDHKFTPLLCEIWD
**FXR-Lig26**	ELTPDQQTLLHFIMDSYNKQRMPQEITNKILKEAFSAEENFLILTEMATNHVQVLVEFTK<br>KLPGFQTLDHEDQIALLKGSAVEAMFLRSAEIFNKKLPAGHSDLLEARIRNSGISDEYIT<br>PMFSFYKSIGELKMTQEEYALLTAIVILSPDRQYIKDREAVEKLQEPLLDVLQKLCKIHQP<br>ENPQHFACLLGRLTELRTFNHHHAEMLMSWAVNDHKFTPLLCEIWD
**FXR-Lig27**	ELTPDQQTLLHFIMDSYNKQRMPQEITNKILKEAFSAEENFLILTEMATNHVQVLVEFTK<br>KLPGFQTLDHEDQIALLKGSAVEAMFLRSAEIFNKKLPAGHSDLLEARIRNSGISDEYIT<br>PMFSFYKSIGELKMTQEEYALLTAIVILSPDRQYIKDREAVEKLQEPLLDVLQKLCKIHQP<br>ENPQHFACLLGRLTELRTFNHHHAEMLMSWAVNDHKFTPLLCEIWD
**FXR-Lig28**	ELTPDQQTLLHFIMDSYNKQRMPQEITNKILKEAFSAEENFLILTEMATNHVQVLVEFTK<br>KLPGFQTLDHEDQIALLKGSAVEAMFLRSAEIFNKKLPAGHSDLLEARIRNSGISDEYIT<br>PMFSFYKSIGELKMTQEEYALLTAIVILSPDRQYIKDREAVEKLQEPLLDVLQKLCKIHQP<br>ENPQHFACLLGRLTELRTFNHHHAEMLMSWAVNDHKFTPLLCEIWD
**FXR-Lig29**	ELTPDQQTLLHFIMDSYNKQRMPQEITNKILKEAFSAEENFLILTEMATNHVQVLVEFTK<br>KLPGFQTLDHEDQIALLKGSAVEAMFLRSAEIFNKKLPAGHSDLLEARIRNSGISDEYIT<br>PMFSFYKSIGELKMTQEEYALLTAIVILSPDRQYIKDREAVEKLQEPLLDVLQKLCKIHQP<br>ENPQHFACLLGRLTELRTFNHHHAEMLMSWAVNDHKFTPLLCEIWD
**FXR-Lig30**	ELTPDQQTLLHFIMDSYNKQRMPQEITNKILKEAFSAEENFLILTEMATNHVQVLVEFTK<br>KLPGFQTLDHEDQIALLKGSAVEAMFLRSAEIFNKKLPAGHSDLLEARIRNSGISDEYIT<br>PMFSFYKSIGELKMTQEEYALLTAIVILSPDRQYIKDREAVEKLQEPLLDVLQKLCKIHQP<br>ENPQHFACLLGRLTELRTFNHHHAEMLMSWAVNDHKFTPLLCEIWD
**FXR-Lig31**	ELTPDQQTLLHFIMDSYNKQRMPQEITNKILKEAFSAEENFLILTEMATNHVQVLVEFTK<br>KLPGFQTLDHEDQIALLKGSAVEAMFLRSAEIFNKKLPAGHSDLLEARIRNSGISDEYIT<br>PMFSFYKSIGELKMTQEEYALLTAIVILSPDRQYIKDREAVEKLQEPLLDVLQKLCKIHQP<br>ENPQHFACLLGRLTELRTFNHHHAEMLMSWAVNDHKFTPLLCEIWD
**FXR-Lig32**	ELTPDQQTLLHFIMDSYNKQRMPQEITNKILKEAFSAEENFLILTEMATNHVQVLVEFTK<br>KLPGFQTLDHEDQIALLKGSAVEAMFLRSAEIFNKKLPAGHSDLLEARIRNSGISDEYIT<br>PMFSFYKSIGELKMTQEEYALLTAIVILSPDRQYIKDREAVEKLQEPLLDVLQKLCKIHQP<br>ENPQHFACLLGRLTELRTFNHHHAEMLMSWAVNDHKFTPLLCEIWD

Structure	Sequence
**FXR-Lig34**	ELTPDQQTLLHFIMDSYNKQRMPQEITNKILKEAFSAEENFLILTEMATNHVQVLVEFTKKLPGFQTLDHEDQIALLKGSAVEAMFLRSAEIFNKKLPAGHSDLLEARIRNSGISDEYITPMFSFYKSIGELKMTQEEYALLTAIVILSPDRQYIKDREAVEKLQEPLLDVLQKLCKIHQPENPQHFACLLGRLTELRTFNHHHAEMLMSWAVNDHKFTPLLCEIWD
**FXR-Lig35**	ELTPDQQTLLHFIMDSYNKQRMPQEITNKILKEAFSAEENFLILTEMATNHVQVLVEFTKKLPGFQTLDHEDQIALLKGSAVEAMFLRSAEIFNKKLPAGHSDLLEARIRNSGISDEYITPMFSFYKSIGELKMTQEEYALLTAIVILSPDRQYIKDREAVEKLQEPLLDVLQKLCKIHQPENPQHFACLLGRLTELRTFNHHHAEMLMSWAVNDHKFTPLLCEIWD
**FXR-Lig36**	ELTPDQQTLLHFIMDSYNKQRMPQEITNKILKEAFSAEENFLILTEMATNHVQVLVEFTKKLPGFQTLDHEDQIALLKGSAVEAMFLRSAEIFNKKLPAGHSDLLEARIRNSGISDEYITPMFSFYKSIGELKMTQEEYALLTAIVILSPDRQYIKDREAVEKLQEPLLDVLQKLCKIHQPENPQHFACLLGRLTELRTFNHHHAEMLMSWAVNDHKFTPLLCEIWD

Table S4 Comparison of the RMSD towards experiments for the top-predicted pose when including all protein structures (globally first) or only the protein structure determined for the particular ligand (self first). The shaded table entries represent poses with an RMSD below 2 Å. The average RMSD and the number of poses with RMSD below 2 Å are also reported. In cases where the shading status changes, an explaining comment is also given, in which "structure *X*" denotes the protein structure taken from the experimental complex with ligand *X*. It can be seen that a hypothetical procedure, which includes only protein structures with ligands of the same type, would have given only one additional correct top-prediction (for ligand 10, for which all the structures with better scores are from complexes of the benzimidazole type), i.e. in total 20 correct poses. It can also be noted that only 9 ligands had the best score for its "own" crystal structure (i.e. the "globally first" equal to the "self first" pose); this mainly reflects the similarity between the ligands.

Ligand	RMSD (Å) globally first	RMSD (Å) self first	Comment
1	0.65	0.65	
2	6.86	3.72	
3	4.92	5.08	
4	0.69	0.69	
5	6.21	7.04	
6	0.42	0.41	
7	1.12	1.28	
8	5.55	5.55	
9	0.44	0.52	
10	9.03	1.70	Better scores for wrong poses with structures 8, 27, 31, 35, and 36
11	9.68	9.84	
12	9.22	2.34	
13	0.38	0.38	
14	0.87	1.67	
15	1.23	1.23	
16	1.74	0.91	
17	1.47	1.59	
18	8.83	1.21	Better scores for wrong poses with 27 other structures
19	1.40	5.01	Correct pose with better score found with structures 22, 28–31, 35, 36
20	0.72	1.68	
21	4.93	6.23	
22	2.15	0.69	Better score for wrong pose with structure 6
23	7.63	6.77	
24	4.86	6.55	
25	0.49	0.49	
26	8.10	1.03	Better score for wrong pose with structure 32
27	1.25	1.33	
28	1.15	0.63	
29	0.50	0.50	
30	7.42	5.54	
31	1.60	1.57	
32	3.96	3.96	
34	11.03	11.15	
35	0.40	0.62	
36	0.66	0.66	
**Avg**	**3.64**	**2.86**	
**# correct**	**19**	**22**	

Table S5 Reliability of the scoring function. The table shows the best score for any docking pose that has an RMSD below 2 Å towards experiment, and the difference between this score and the "globally first" score given in Table 1. The ligands are sorted according to decreasing score difference, and the table only shows the 29 ligands for which correct poses were found. A difference of zero means that there is an acceptable pose that has the best score; this is not necessarily the "globally best" pose from Table 1.

Ligand	Score (kcal/mol)	
	best correct score	difference
18	-9.0	3.4
12	-9.7	1.6
23	-10.2	1.5
24	-11.9	1.2
10	-9.8	1.1
8	-11.0	0.4
21	-12.3	0.4
30	-11.0	0.4
26	-12.5	0.2
22	-12.0	0.1
1	-10.7	0
4	-12.0	0
6	-12.2	0
7	-13.9	0
9	-13.2	0
13	-14.9	0
14	-12.0	0
15	-12.0	0
16	-10.6	0
17	-11.2	0
19	-12.2	0
20	-12.9	0
25	-13.3	0
27	-13.7	0
28	-13.0	0
29	-12.9	0
31	-12.3	0
35	-13.5	0
36	-13.9	0

MM-PBSA analysis of MD trajectories

We selected some example ligands to investigate whether MM-PBSA estimation of binding free energies provides sufficient statistical significance to discriminate between various binding poses and, in that case, whether it ranks the poses in agreement with their RMSD towards experiment.

Methods

The MM-PBSA calculations were performed using the stand-alone `g_mmpbsa` tool.The binding free energy was calculated for each system using 250 snapshots from a 30 ns MD simulation with the first 5 ns discarded, i.e. with a stride of 0.1 ns. The MM-PBSA energy was computed for each snapshot as a sum of three terms:

$$\Delta G_{\mathrm{bind}}^{\mathrm{MM\text{-}PBSA}} = E_{\mathrm{MM}}^{\mathrm{int}} + \Delta G_{\mathrm{PB}} + \Delta G_{\mathrm{np}} \ . \tag{1}$$

Here, $E_{\mathrm{MM}}^{\mathrm{int}}$ is the MM interaction energy (sum of electrostatics and van der Waals energy) between the protein and the ligand. ΔG_{PB} is the change in Poisson-Boltzmann solvation energy upon ligand binding, with a solute dielectric constant of 1, a water dielectric of 80, a solvent probe radius of 1.4 Å, the *smol* model for construction of the cavity, Bondii radii, and otherwise default parameters of the `g_mmpbsa` software. Finally, ΔG_{np}, the nonpolar solvation energy, was calculated using the following equation:

$$\Delta G_{\mathrm{np}} = \gamma \Delta(SASA) + \delta \ , \tag{2}$$

where $\gamma = 5.43$ kcal mol^{-1} nm^{-2}, $\delta = 0.92$ kcal/mol, and the SASA was calculated using a probe radius of 1.4 Å and default parameters of the `g_mmpbsa` software. Note that only relative binding free energies can be evaluated in this case owing to the absence of the entropy term from the calculation.

Error analysis

The values reported in Table S6 are the averages (μ) and standard deviations (σ) over the N snapshots. The standard error of the mean (SE) is calculated by assuming normally distributed values while taking into account the statistical inefficiency (s) of the samples, i.e. as

$$SE = \frac{\sigma}{\sqrt{N/s}} \tag{3}$$

The SE is an estimate of the statistical uncertainty and gives a lower bound to what energy differences between poses that are meaningful to discuss. More precisely, we apply Welch's unequal variances t-test to estimate the probability (p) for the mean values of two distributions being equal.

Unfortunately, the systematic errors, primarily caused by the simplified treatment of solvation effects, are much more difficult to estimate. In our analysis, we are not concerned with the errors in the absolute binding free energy, nor the errors in the relative binding free energy between various ligands. Instead, we are only concerned with the systematic shift between various binding poses for the same ligand. To exemplify that there still is a problem, we note that the three MD-simulated poses for ligand 30 have completely different electrostatic contributions, ranging from 2 to 125 kcal/mol, and a similar range for the PB contributions, which are

known to roughly counterbalance the electrostatic contribution, especially for charged ligands. Evidently, the electric field surrounding the ligand is very different in the three poses, and thus one can not rely on cancellation of error between the various poses, with regards to the treatment of electrostatics and solvation. The real uncertainty is therefore much larger than the SE (which is only 1–4 kcal/mol), as the latter reflects the fluctuations around a single well-defined pose with no significant changes in the electrostatic surroundings.

Our interpretation of the results in Table S6 is that among the charged ligands (30 and 36), all comparisons except that between the "best" and experimental poses of ligand 36, probably have much larger uncertainty than indicated by the SE, possibly greater than 10 kcal/mol. For ligand 32, the experimental pose has rather different electrostatics and thus the uncertainty in the difference towards the two other poses is larger than indicated by the SE, although probably not as large as for the charged ligands. For ligand 25, the uncertainty in the difference between the "first" and the other poses is also somewhat underestimated by the SE.

Results

A summary of the MM-PBSA results in relation to the RMSD from experiment are given in Table S7. For ligands 20, 24, 25, and 36, the ensemble started from the "best" pose is (in terms of RMSD) almost identical to that started from the experimental pose, and this is largely confirmed by the MM-PBSA results, with a maximum discrepancy of 1.4 kcal/mol (which is still somewhat larger than expected from the SEs).

For ligands 20, 24, and 25, the "first" pose has the least negative binding free energy, in agreement with its higher RMSD towards experiment, and contrasting to the prediction by the scoring function. The p-values are 0.0008, 0.0001, and 0.26, respectively, indicating that the energy difference relative to the "Exp" pose is significant in case of ligands 20 and 24, but not for ligand 25 (the apparent significancy relative to the "best" pose is not to be trusted, because the "best" and "Exp" poses are almost identical). For ligand 32, all three poses are different, and again the Exp pose has the most negative binding free energy, in agreement with the crystal structure (p-values 0.0001 and 0.0001 for the first and best pose, respectively, relative to the Exp pose). The "first" and "best" poses are mis-ranked according to the RMSD towards experiment, but the energy difference is rather small and the real ranking might not be reflected by the RMSD anyway.

For the charged ligands, the energy ranking also agrees with the RMSD, both for the three different poses of ligand 30, and for the two poses of ligand 36. However, although the energy differences are very large in magnitude, it should be noted that the uncertainties in these values are very difficult to estimate owing to the electrostatic effects discussed above. Thus, we can not reliably classify the differences as significant.

In conclusion, this limited investigation suggests that the MM-PBSA method is useful for predicting the relative binding free energies between several poses of the same ligand, even though the actual binding free energies are much less accurate. The method is apparently more reliable for uncharged ligands, for which the systematic errors in the treatment of electrostatics tend to cancel out between the various poses. It is important to mention that standard deviations from ensembles generated by a single MD simulation are often underestimations, and thus more reliable results would have been obtained by collecting snapshots from multiple shorter MD simulations with different initial velocities [51].

Table S6 Detailed MM-PBSA results for the 18 analyzed simulations. For each simulation, started either from the *First*, *Best*, or experimental (Exp) pose, we report the Van der Waals interaction energy (vdw), the electrostatic interaction energy (ele), the polar solvation energy (PB), the non-polar solvation energy (np), and the sum of these terms (Total). For each quantity, the mean (μ) and standard deviation (σ) over the ensemble is given, along with the standard error of the mean (SE) for the total energy. All values are in kcal/mol.

	First		Best		Exp	
	μ	σ	μ	σ	μ	σ
**Ligand 20**						
vdw	-67.3	2.8	-72.1	2.9	-70.9	2.9
ele	-10.6	2.1	-16.2	2.4	-12.7	3.4
PB	38.5	3.8	47.0	2.3	42.8	2.8
np	-6.3	0.2	-6.5	0.2	-6.3	0.2
Total	-45.7	4.1	-47.7	3.3	-47.1	3.2
SE		0.2		0.3		0.3
**Ligand 24**						
VdW	-59.5	2.9	-63.9	2.6	-63.9	2.5
Elec	-9.2	3.3	-8.5	2.2	-9.1	2.2
PB	37.1	2.6	39.3	2.1	39.2	2.3
Nonpol	-5.9	0.2	-5.8	0.2	-5.8	0.2
Total	-37.7	3.4	-38.9	3.4	-39.6	3.1
SE		0.5		0.3		0.2
**Ligand 25**						
VdW	-66.6	2.9	-68.5	2.7	-67.5	2.7
Elec	-22.7	4.6	-14.6	2.7	-13.5	2.5
PB	53.9	3.7	46.0	2.6	45.3	2.3
Nonpol	-5.9	0.2	-6.0	0.2	-6.0	0.2
Total	-41.3	3.1	-43.1	3.5	-41.7	3.1
SE		0.3		0.3		0.3
**Ligand 30**						
VdW	-66.1	3.2	-71.6	3.7	-73.0	3.9
Elec	125.4	9.0	48.5	15.4	1.9	17.4
PB	-77.2	8.9	-6.8	16.1	23.9	11.0
Nonpol	-6.8	0.2	-6.6	0.2	-6.4	0.2
Total	-24.6	5.6	-36.4	6.1	-53.7	10.2
SE		0.7		0.6		3.6
**Ligand 32**						
VdW	-68.3	2.6	-67.3	2.8	-66.9	3.1
Elec	-14.1	3.3	-10.8	4.9	-26.2	4.8
PB	51.0	3.4	47.6	5.0	55.6	3.4
Nonpol	-6.4	0.2	-6.2	0.2	-6.1	0.2
Total	-37.8	4.1	-36.7	4.8	-43.7	4.3
SE		0.5		0.7		0.5
**Ligand 36**						
VdW	-73.7	3.0	-73.3	3.2	-75.7	3.5
Elec	146.0	10.4	21.6	14.4	25.8	9.7
PB	-85.5	10.2	18.2	11.7	16.4	9.3
Nonpol	-6.8	0.2	-6.7	0.2	-6.6	0.2
Total	-20.0	6.8	-40.1	6.2	-40.1	6.0
SE		0.9		0.8		1.3

Table S7 Summary of the MM-PBSA results. For each considered starting pose, the RMSD (in Å) for the main cluster towards experiment, the binding free energy $\Delta G_{\text{MM-PBSA}}$ (in kcal/mol), and the standard error of the mean (SE) for this estimate. SE values that are considered significant underestimates of the real uncertainty are put in brackets (see section Error analysis).

Ligand	Pose	RMSD	$\Delta G_{\text{MM-PBSA}}$	SE
20	First	6.31	-45.7	0.2
	Best	0.67	-47.7	0.3
	Exp	0.70	-47.1	0.3
24	First	6.39	-37.7	0.5
	Best	0.64	-38.9	0.3
	Exp	0.69	-39.6	0.2
25	First	1.73	-41.3	(0.3)
	Best	0.46	-43.1	0.3
	Exp	0.47	-41.7	0.3
30	First	6.45	-24.6	(0.7)
	Best	1.62	-36.4	(0.6)
	Exp	0.78	-53.7	(3.6)
32	First	4.29	-37.8	0.5
	Best	2.27	-36.7	0.7
	Exp	0.80	-43.7	(0.5)
36	First	7.82	-20.0	(0.9)
	Best	0.90	-40.1	0.8
	Exp	0.92	-40.1	1.3

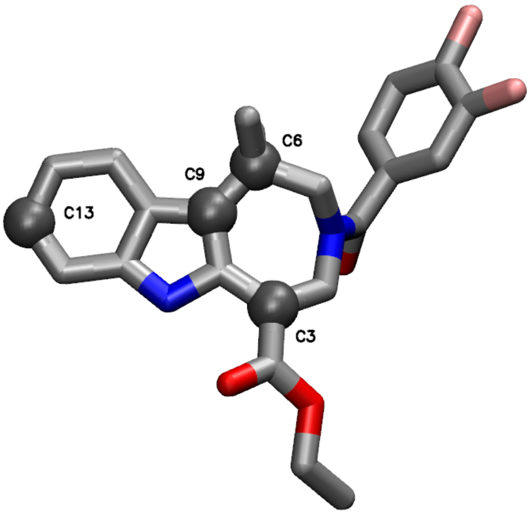

Fig. S1 Picture of ligand 5 with labelling of the four carbon atoms that were used for defining the rotational CVs.The first dihedral was [Met-294 – Leu-291 – C9 – C13], the second was [Ile-277 – Ile-273 – C9 – C13], and third was [Ile-339 – Ser-336 – C6 – C3], where the first two labels in each bracket represent C_α atoms of the protein, and the last two labels represent carbon atoms of the ligand.

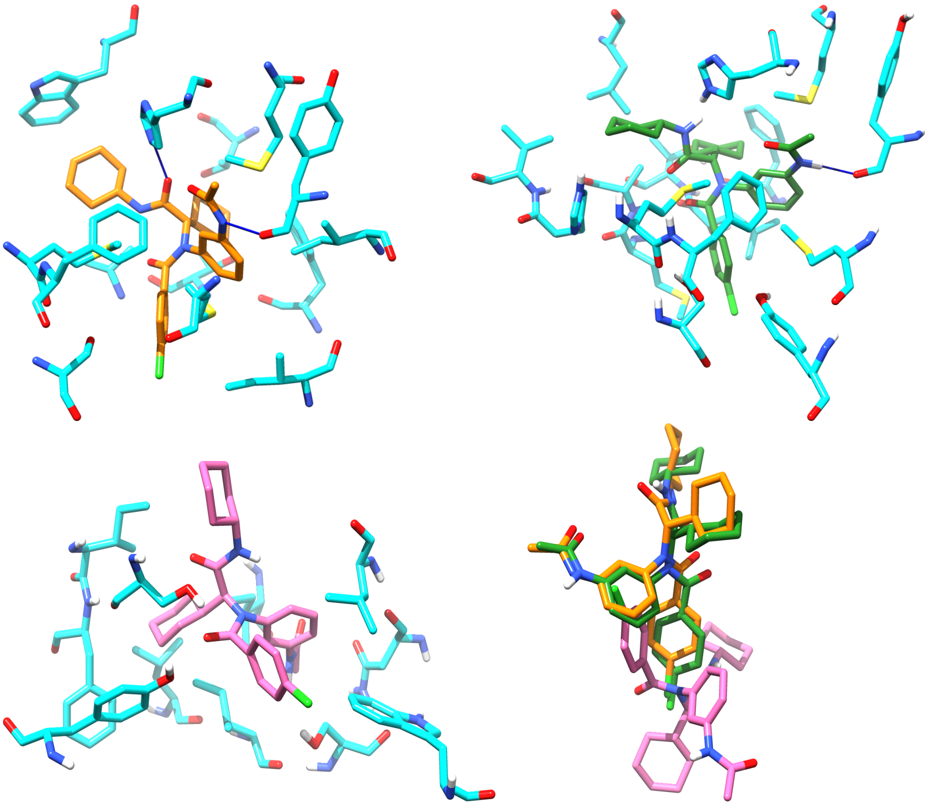

Fig. S2 Comparison of the different poses for ligand 18. *Top left:* Experimental pose. *Top right:* Best docking pose found (RMSD 1.2 Å), with the main difference being the rotated amide group causing a lost hydrogen bond with His-451. *Bottom left:* Top-scored ("first") docking pose (RMSD 8.8 Å) with quite different interactions. *Bottom right:* Overlay of the three poses (using the same coloring of the ligand).

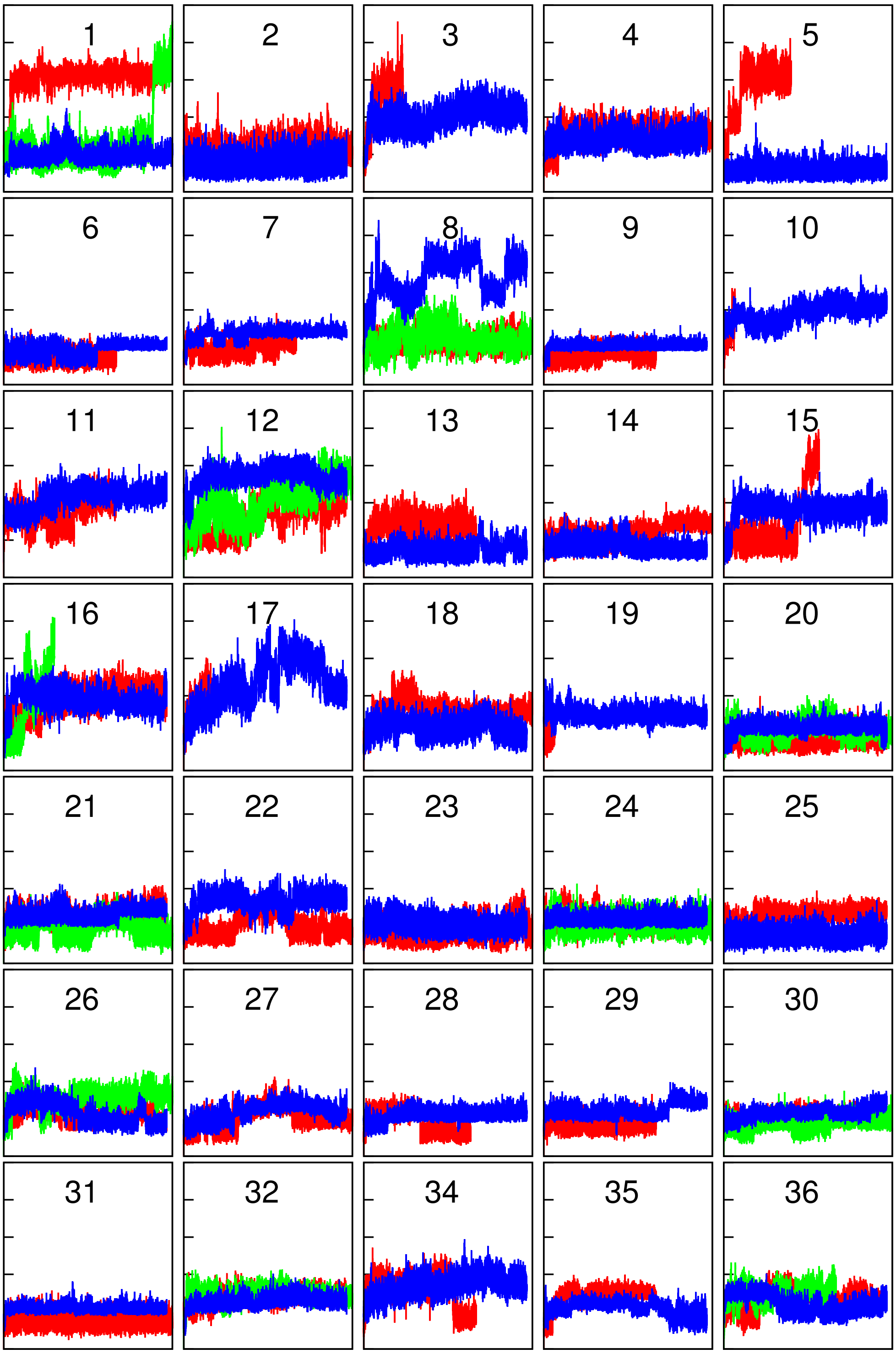

Fig. S3 Ligand RMSD towards the starting structure as a function of simulation time for the MD simulations performed for each ligand: starting from the first docking pose (red line), the best docking pose (green line), and the experimental pose (blue line). The scale on the y axis goes from 0 to 5 Å. The scale on the x axis goes from 0 to 30 ns (many simulations were run until 50 ns but visual inspection ensured that no significant events occured in the 30–50 ns interval).

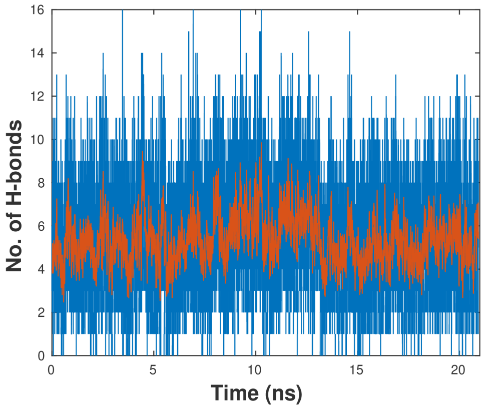

A

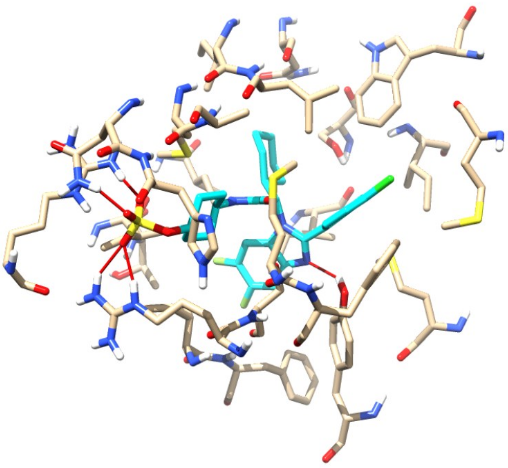

B

Fig. S4 Hydrogen bond analysis for ligand 35 explaining its lack of conformational variation in the RMD simulations. A) Total number of protein–ligand hydrogen bonds during the RMD simulation. The running average is marked with orange. B) 3D representation of the ligand (cyan)–protein hydrogen bonds (red lines) generated using UCSF Chimera.

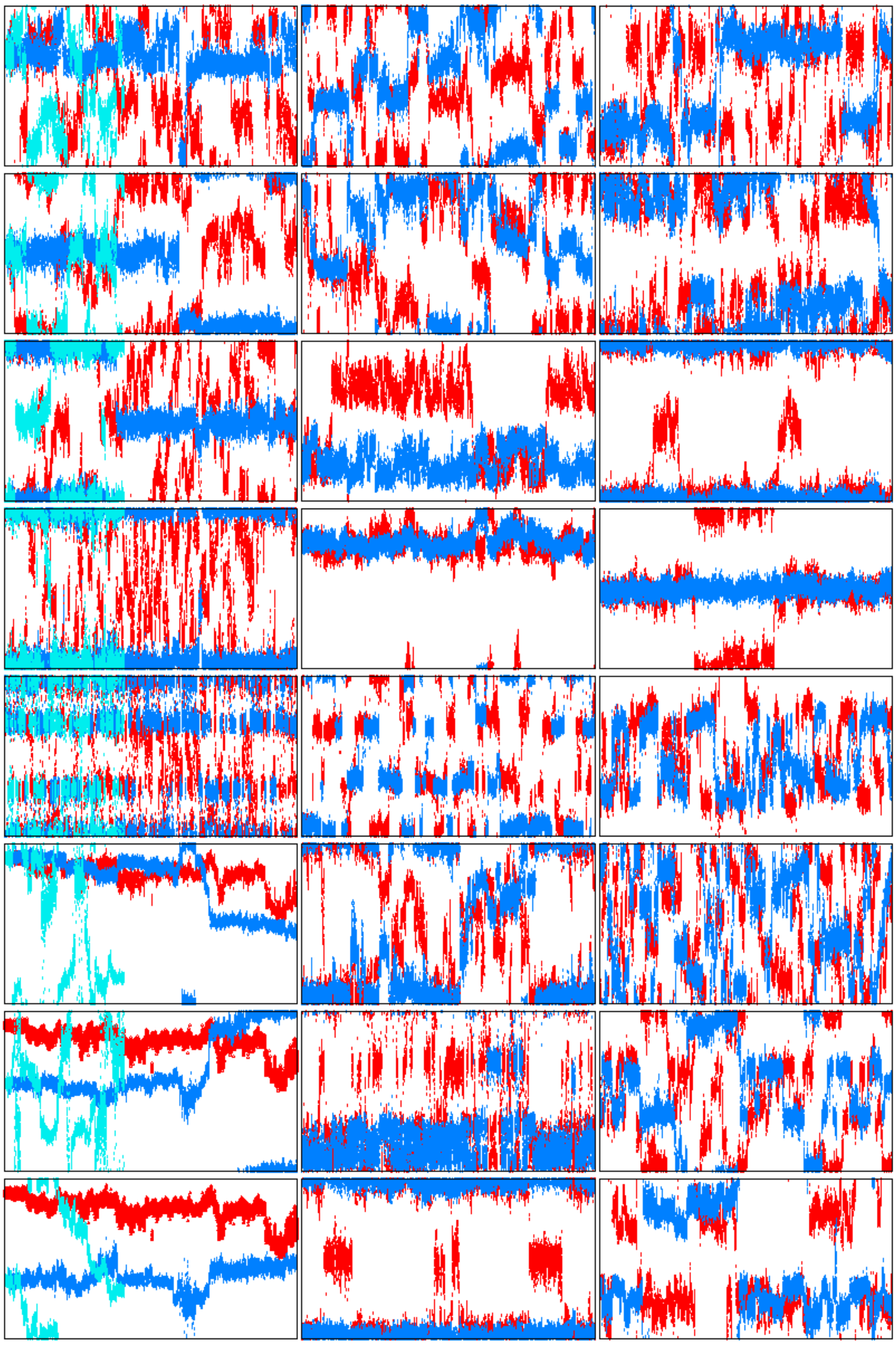

Fig. S5 Fluctuation of the ligand dihedrals in RMD simulations using only the ligand dihedrals as CVs (red), using the ligand dihedrals and side-chain dihedrals as CVs (blue), and using the ligand dihedrals and orientational CVs (cyan). The left, central, and right columns of panels show results for ligand 5, 13, and 15, respectively. Each panel shows how one of the ligand dihedrals varies with time. For ligand 5, which have only 5 rotable ligand dihedrals, the bottom three panels show the orientational CVs instead (which were only monitored for the blue and red simulations but included as CVs in the cyan simulation). The scale on the x axis always goes from 0 to 20 ns. The scale on the y axis always goes from −180° to +180° and is periodic so that the upper endpoint of the axis represents the same conformation as the lower endpoint of the axis.

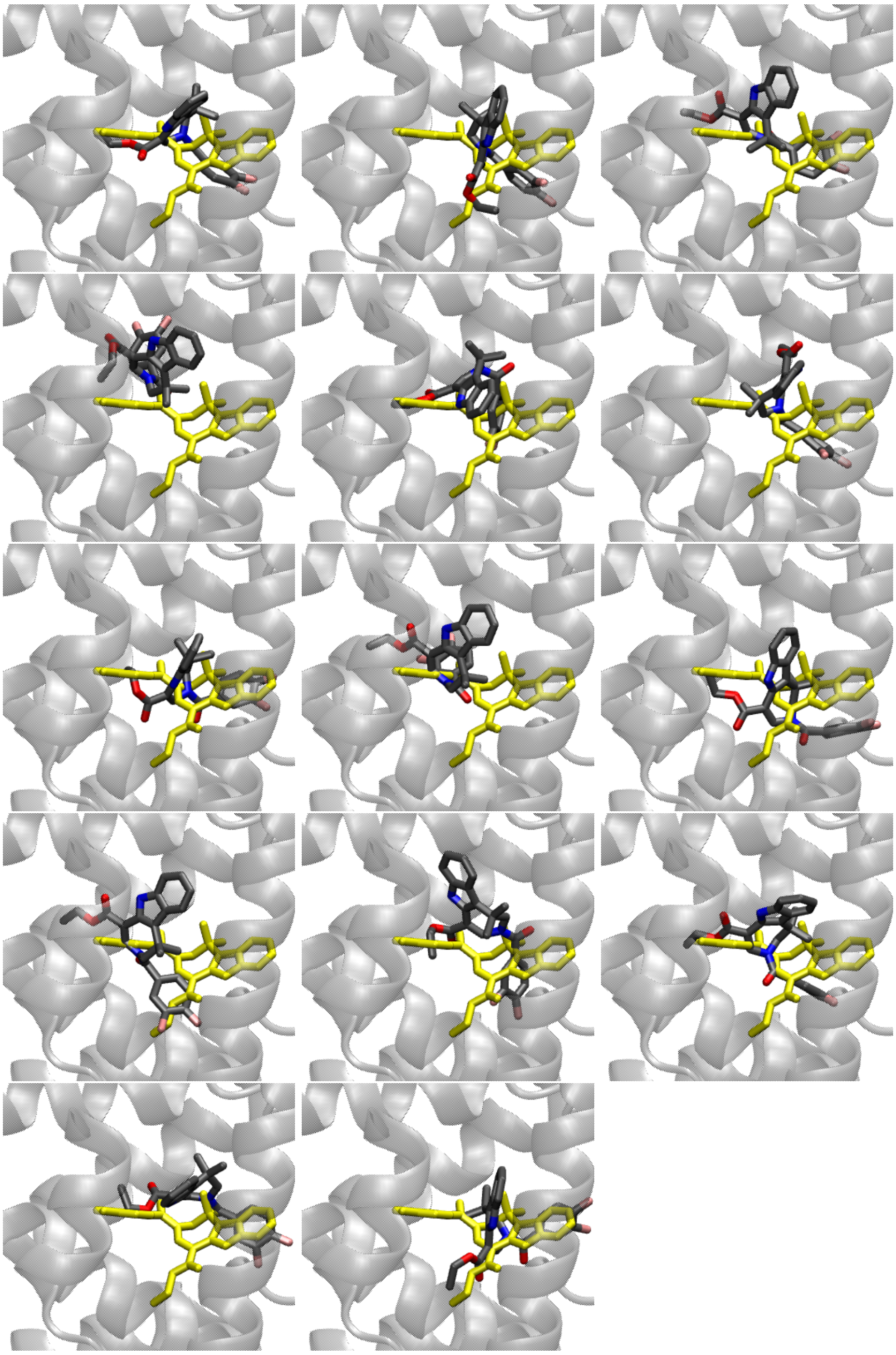

Fig. S6 All significant clusters obtained from the RMD simulation of ligand 5 using only the ligand dihedrals as CVs. The experimental pose is shown in yellow.

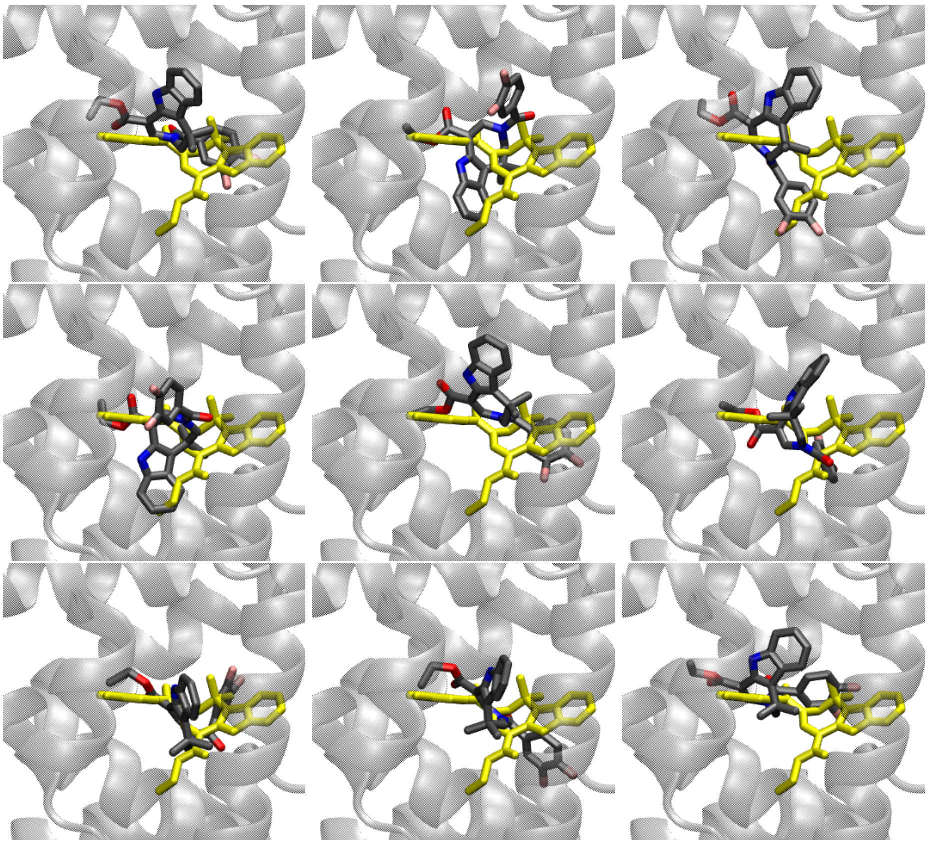

Fig. S7 All significant clusters obtained from the RMD simulation of ligand 5 using the ligand dihedrals *and side-chain dihedrals* as CVs. The experimental pose is shown in yellow.

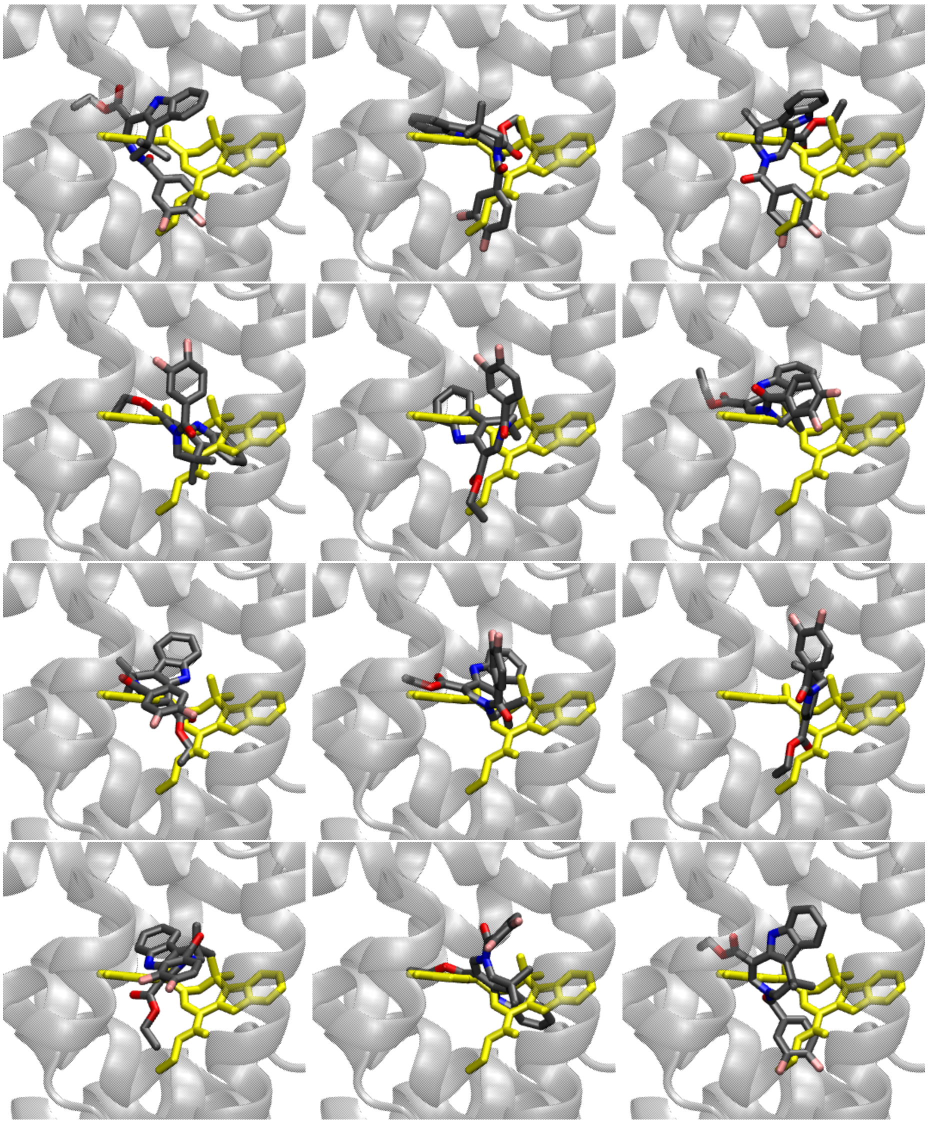

Fig. S8 All significant clusters obtained from the RMD simulation of ligand 5 using the ligand dihedrals *and rotation-promoting dihedrals* as CVs. The experimental pose is shown in yellow.
